# Unified model for singlet fission within a non-conjugated covalent pentacene dimer

**DOI:** 10.1038/ncomms15171

**Published:** 2017-05-18

**Authors:** Bettina S. Basel, Johannes Zirzlmeier, Constantin Hetzer, Brian T. Phelan, Matthew D. Krzyaniak, S. Rajagopala Reddy, Pedro B. Coto, Noah E. Horwitz, Ryan M. Young, Fraser J. White, Frank Hampel, Timothy Clark, Michael Thoss, Rik R. Tykwinski, Michael R. Wasielewski, Dirk M. Guldi

**Affiliations:** 1 Department of Chemistry and Pharmacy & Interdisciplinary Center for Molecular Materials (ICMM), Friedrich-Alexander-Universität Erlangen-Nürnberg (FAU), Egerlandstrasse 3, 91058 Erlangen, Germany; 2 Department of Chemistry and Pharmacy & Interdisciplinary Center for Molecular Materials (ICMM), Friedrich-Alexander-Universität Erlangen-Nürnberg (FAU), Henkestrasse 42, 91054 Erlangen, Germany; 3 Department of Chemistry and Argonne-Northwestern Solar Energy Research (ANSER) Center, Northwestern University, Evanston, Illinois 60208-3113, USA; 4 Institute for Theoretical Physics & Interdisciplinary Center for Molecular Materials (ICMM), Friedrich-Alexander-Universität Erlangen-Nürnberg (FAU), Staudtstrasse 7/B2, 91058 Erlangen, Germany; 5 Rigaku Europe, Unit B6, Chaucer Business Park, Watery Lane, Kemsing, Sevenoaks TN15 6QY, UK; 6 Department of Chemistry and Pharmacy & Computer-Chemistry-Center (CCC), Friedrich-Alexander-Universität Erlangen-Nürnberg, Nägelsbachstrasse 25, 91052 Erlangen, Germany

## Abstract

When molecular dimers, crystalline films or molecular aggregates absorb a photon to produce a singlet exciton, spin-allowed singlet fission may produce two triplet excitons that can be used to generate two electron–hole pairs, leading to a predicted ∼50% enhancement in maximum solar cell performance. The singlet fission mechanism is still not well understood. Here we report on the use of time-resolved optical and electron paramagnetic resonance spectroscopy to probe singlet fission in a pentacene dimer linked by a non-conjugated spacer. We observe the key intermediates in the singlet fission process, including the formation and decay of a quintet state that precedes formation of the pentacene triplet excitons. Using these combined data, we develop a single kinetic model that describes the data over seven temporal orders of magnitude both at room and cryogenic temperatures.

## Abstract (web summary)

Singlet fission is an important process occurring in solar cells, however the mechanism is not well understood. Here the authors reveal intermediates during singlet fission of a non-conjugated pentacene dimer, developing a single kinetic model to describe the data over seven temporal orders of magnitude at room and cryogenic temperatures.

Innovative strategies are necessary to overcome the Shockley–Queisser limit^1^, which places the maximum solar conversion efficiency for a single p–n junction at ∼33%. One such strategy is singlet fission (SF), in which a high-energy, photogenerated singlet exciton (S_1_) in an organic material is rapidly down-converted into two low-energy, triplet excitons (T_1_+T_1_) in a spin-allowed process, provided that the state energies fulfil the requirement: *E*(S_1_)≥2*E*(T_1_) (refs 2, 3). The SF rate depends on the free energy of the process and the electronic coupling matrix elements between the chromophores for the particular states involved in this process^2^,^3^. Several families of molecular building blocks have been identified that fulfil the energetic requirements for efficient SF, including polyacenes^4^,^5^,^6^,^7^,^8^,^9^, diphenylisobenzofurans^10^, carotenoids^11^,^12^, rylenes^13^ and other chromophores^14^, although the scope of SF molecules still remains rather limited, as discussed in reviews by Smith and Michl^2^,^3^.

SF can occur by two general mechanisms: one that directly couples the photogenerated initial ^1^(S_1_S_0_) state to a multi-exciton ^1^(T_1_T_1_) state by a two-electron process, and the other, in which the conversion of ^1^(S_1_S_0_) into ^1^(T_1_T_1_) is facilitated by coupling to higher-lying charge transfer (CT) states^2^. It is also possible that the ^1^(T_1_T_1_) state can undergo spin evolution to yield the ^5^(T_1_T_1_) quintet state, which upon spin decoherence can produce two non-interacting triplet states (T_1_+T_1_)^15^,^16^. In addition, it has recently been suggested that the two separated T_1_ states can be populated directly from the ^1^(S_1_S_0_) state without participation of any intermediate electronic states^12^. Given the number of states potentially involved in SF and their relationships to one another, pentacene dimers have emerged as an important model for exploring SF^8^,^17^,^18^,^19^,^20^. They offer the opportunity to fine-tune the electronic coupling between neighbouring pentacenes by means of distance and effective π-conjugation between them. As a result, intramolecular SF rates vary from 2.5 × 10^12^ s^−1^ in dimers with orthogonal pentacenes to 1.8 × 10^9^ s^−1^ in bent dimers^21^,^22^, while the SF rates in polycrystalline pentacenes are generally >10^12^ s^−1^ (refs 4, 23). Moreover, it has been hypothesized that the electronic coupling between the pentacenes is critical to render CT-state-mediated SF energetically accessible. While no evidence for CT-mediated SF was observed in pentacene dimers linked directly or using *p*-phenylene oligomers^24^,^25^, work on π-conjugated and cross-conjugated pentacene dimers^26^,^27^ has shown that effective π-conjugation enables fast, CT-state-mediated SF.

To date, all reported pentacene dimers have been designed with conjugated spacers. We report here a pentacene dimer linked by a rigid, non-conjugated 1,3-diethynyladamantyl spacer (NC, Fig. 1). The pentacenes in NC are coupled just strongly enough to allow efficient SF before any other excited state processes occur, but weakly enough that the ^1^(T_1_T_1_) state formed by SF can undergo spin-mixing to form the ^5^(T_1_T_1_) on a timescale sufficiently slow to allow time-resolved electron paramagnetic resonance (TREPR) spectroscopy to observe both its formation and decay into independent triplet states (T_1_+T_1_). This contrasts with recent observations of quintet states formed by SF in tetracene films and conjugated pentacene dimers^15^,^16^, and allows us to use complementary time-resolved optical and TREPR spectroscopy to observe the intermediates and kinetics for the entire ^1^(S_1_S_0_) 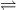 ^1^(T_1_T_1_) 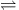 ^5^(T_1_T_1_)→(T_1_+T_1_) sequence. Using these combined data, we develop a single kinetic model that describes the data over seven temporal orders of magnitude both at room and cryogenic temperatures.

## Results

### Synthesis

**Figure 2:**
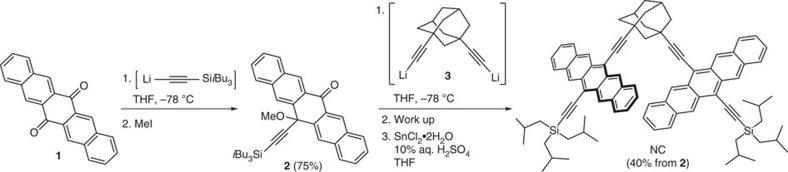
Synthesis of NC. Detailed steps for the preparation of NC.

Non-conjugated, rigid pentacene dimer NC was synthesized by the stepwise desymmetrization of 6,13-pentacenequinone **1**—Fig. 2. Lithiated tri-*iso*butylsilylacetylene was added to a suspension of **1** in THF at low temperature, and after reaction for 5 h, the reaction was quenched by the addition of MeI to afford ketone **2** in 75% yield. 1,3-Diethynyladamantane was lithiated by a reaction with LiHMDS in THF at low temperature to provide **3**, and ketone **2** was then added to the solution of **3**. After quenching of the reaction by the addition of water, the resulting intermediate was isolated and carried on directly to Sn(II)-mediated reductive aromatization to give pentacene product NC. The crude material was purified by column chromatography to give analytically pure NC in 40% yield over the two steps from **2**. As a solid, the dimer NC is stable to air and moisture under normal laboratory conditions and shows a decomposition point of 364 °C (onset 282 °C, peak 364 °C) as measured by differential scanning calorimetry. The dimer has good solubility in typical organic solvents (4 mg ml^−1^) such as THF, CH_2_Cl_2_, CHCl_3_ and toluene, but NC slowly decomposes over several days in solutions under exposure to air and light (Supplementary Methods 1). The known pentacene monomer 6,13-bis(tri-*iso*butylsilylethynyl)pentacene (TIBS) served as a reference^28^.

### Time-resolved optical spectroscopy

**Figure 3:**
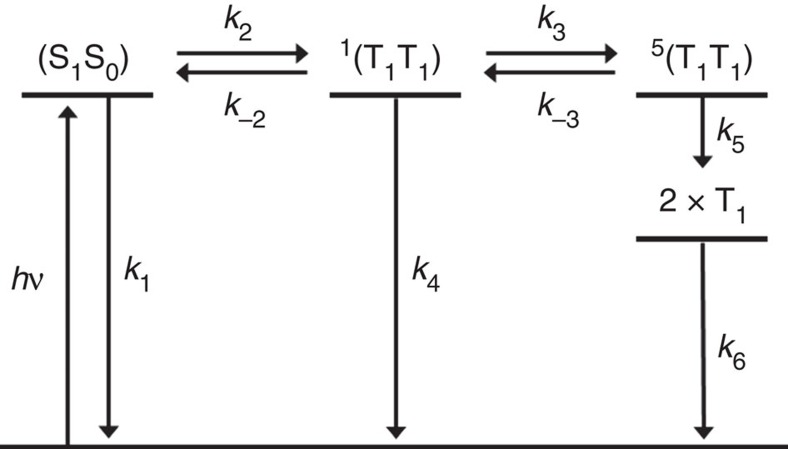
Kinetic model. Kinetic model used to fit both the transient optical absorption and TREPR data for NC in both benzonitrile at 295 K and butyronitrile at 105 K.

Femtosecond and nanosecond transient absorption (fsTA and nsTA) measurements were used to probe the initial SF events in NC by irradiating into the low-energy absorption features in the range of 590–656 nm (Supplementary Figs 8 and 9), and were analysed by both multi-wavelength and global analysis using the kinetic model illustrated in Fig. 3. The model was established using the rate constants obtained from fsTA, nsTA and TREPR spectroscopy on NC in butyronitrile at 105 K. Specifically, the TREPR spectra allow for tracking the time evolution of the ^1^(T_1_T_1_), ^5^(T_1_T_1_) and (T_1_+T_1_) spin states independently following SF, all of which present very similar optical spectra. The fsTA, nsTA and TREPR spectral assignments were then used to formulate the kinetic model in Fig. 3 used to fit the measured rate constants. This model was then applied to fit the fsTA and nsTA data obtained at 295 K (see analysis below and Supplementary Methods 3).

**Figure 4:**
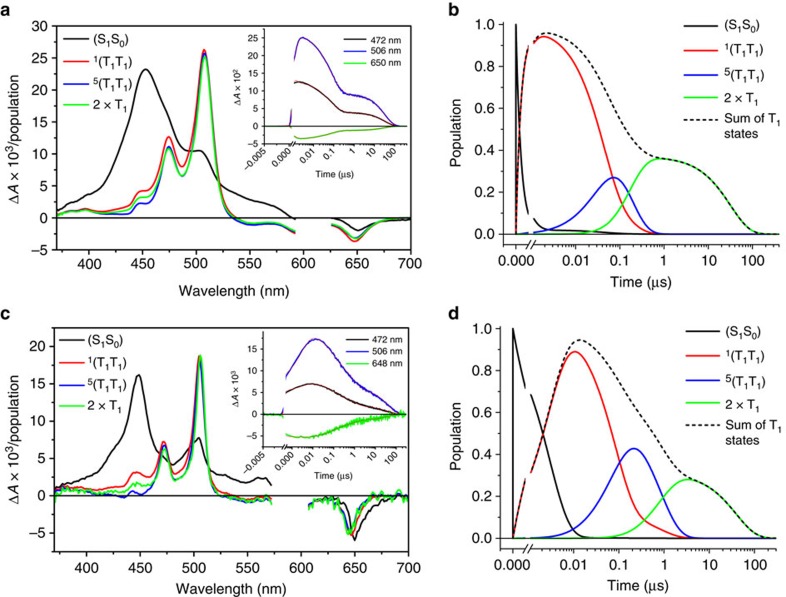
Transient optical absorption data. (**a**) Species-associated transient absorption difference spectra for NC obtained at 295 K in benzonitrile using the kinetic model given in Fig. 3. Inset: single wavelength kinetics and fits to the data. (**b**) Population kinetics for each species. (**c**) Species-associated transient absorption difference spectra for NC obtained at 105 K in butyronitrile using the kinetic model given in Fig. 3. Inset: single wavelength kinetics and fits to the data. (**d**) Population kinetics for each species. The total number of T_1_ states per S_1_ created is twice the population of the T_1_ species indicated in **b**,**d**. Multiplying the species-associated spectra in **a**,**c** by the corresponding species populations in **b**,**d**, respectively, yield the complete Δ*A* versus time and wavelength data set.

The typical singlet excited state characteristics of pentacene derivatives appear within the instrument response function upon either 610/656 nm photoexcitation of NC at 295 K in benzonitrile or 590/610 nm photoexcitation at 105 K in butyronitrile. The species-associated spectra at 295 and 105 K are shown in Fig. 4a,c, respectively, while the raw data are shown in Supplementary Figs 10–13, respectively. For NC, the 652 nm minimum intensifies throughout the transformation into a triplet excited state, as the long wavelength ground-state absorption decreases. This increase in ground-state bleaching indicates a multiplication of excited states, which suggests that triplet state formation following excited singlet state deactivation of NC is dominated by SF rather than spin-orbit-induced intersystem crossing (ISC).

The formation of ^1^(T_1_T_1_) dominates the decay of ^1^(S_1_S_0_) and occurs with *k*_2_=2.4±0.1 × 10^9^ s^−1^ at 295 K and *k*_2_=3.3±0.9 × 10^8^ s^−1^ at 105 K (Table 1). In contrast, the S_1_ state of the TIBS monomer decays much more slowly with *k*=9.5±0.7 × 10^7^ s^−1^ in benzonitrile at 295 K (Supplementary Fig. 14). While the optical spectra of the ^1^(T_1_T_1_), ^5^(T_1_T_1_) and (T_1_+T_1_) states are all very similar, the spectrum of ^1^(T_1_T_1_) shows a modest enhancement in absorption between 450 and 500 nm relative to the other states involving T_1_. Importantly, the transient differential absorption spectra are in good agreement with that of the NC pentacene triplet found in triplet–triplet sensitization experiments using anthracene as a triplet sensitizer (Supplementary Fig. 15).

The uncorrelated triplet states decay in *k*_6_=3.1±0.1 × 10^4^ s^−1^ and 2.5±0.1 × 10^4^ s^−1^ at 295 and 105 K, respectively, which is very similar to the rate for the TIBS monomer reference, where T_1_ decays with *k*=2.9±0.5 × 10^4^ s^−1^ (Supplementary Fig. 14).

The state populations obtained from the global analyses at 295 and 105 K in Fig. 4b,d, respectively, show that the ^1^(T_1_T_1_) yield is 188% at 295 K and 178% at 105 K, while that of the uncorrelated triplets is 72% at 295 K and 56% at 105 K. The triplet quantum yields of NC are far larger than the very low (<1%) triplet yield of the TIBS monomer reference in de-oxygenated benzonitrile that results from the spin-orbit-induced ISC mechanism (Supplementary Fig. 14).

### TREPR spectroscopy

**Figure 5:**
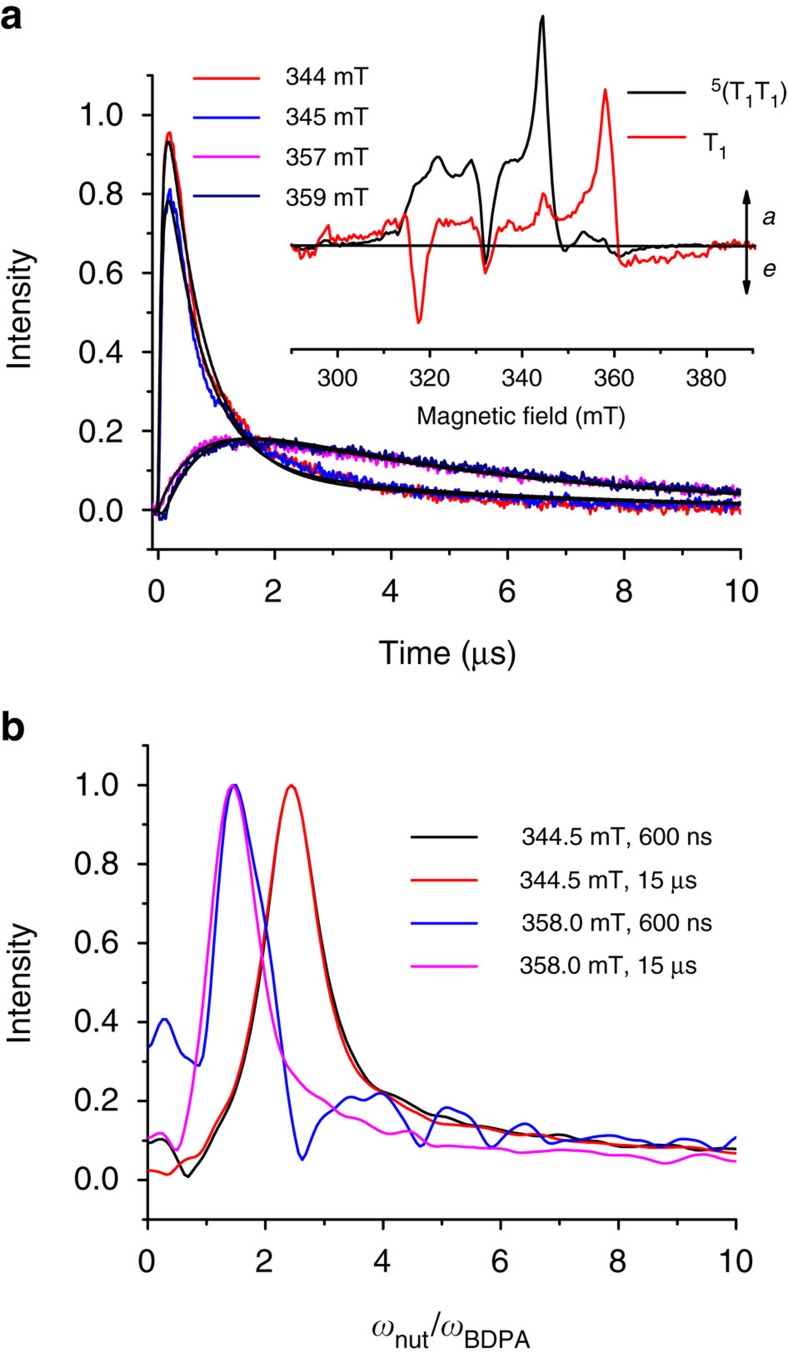
Transient EPR data. (**a**) TREPR kinetics for the formation and decay of the ^5^(T_1_T_1_) and (T_1_+T_1_) states for NC at 105 K in butyronitrile measured at the indicated magnetic field values corresponding to the species-associated spectra of the ^5^(T_1_T_1_) and separated T_1_ states shown in the inset. Black lines superimposed on the kinetic traces are the fits to the data using the model described in Fig. 3. (**b**) Echo-detected transient nutation spectra of NC collected at 5 K in butyronitrile following photoexcitation at 640 nm with a 7 ns, 3 mJ laser pulse. The frequency axis was normalized to the nutation frequency of an *S*=½, BDPA, radical standard collected under the same experimental conditions.

As mentioned above, the results from TREPR spectroscopy are critical to developing the unified kinetic model depicted in Fig. 3. TREPR spectra of NC were acquired in butyronitrile at 105 K following photoexcitation with a 7 ns, 640 nm laser pulse, and are shown in the inset to Fig. 5a, while the TREPR kinetics at four magnetic field values are shown in Fig. 5a. A narrow spin-polarized quintet spectrum appears with *k*_3_=6.7±0.1 × 10^6^ s^−1^ with lines having a (*a,e,e,a,a,e*) (*a*=enhanced absorption, *e*=emission, low-to-high magnetic field) spin polarization pattern. It decays with *k*_5_=6.7±0.1 × 10^5^ s^−1^ into a broader triplet spectrum, which also has a (*a,e,e,a,a,e*) polarization pattern. Interestingly, this polarization pattern is generally characteristic of triplet excited states formed from charge recombination of radical ion pairs produced by the radical-pair intersystem-crossing mechanism^29^, wherein only the T_0_ spin sublevel of the radical ion pair at high magnetic field is populated. The optical transient absorption experiments give no indication for the presence of a sufficiently long-lived charge-separated radical ion pair state in NC. Instead, SF occurs, and the TREPR spectra suggest a mechanism in which the initially formed ^1^(T_1_T_1_) mixes with ^5^(T_1_T_1_) via the *m*_s_=0 state to yield the observed spin polarization pattern^15^,^16^. This is followed by the formation of a pair of independent T_1_ states, whose initial spin polarization preserves that of the precursor quintet state. The spin polarization of the triplet states relaxes with *k*_rlx_=1.3±0.1 × 10^5^ s^−1^, while the overall triplet population decays with *k*_6_=2.5±0.1 × 10^4^ s^−1^, as determined by transient optical absorption spectroscopy.

The zero-field splitting parameter, *D*, which monitors the average distance between the unpaired spins^30^, can be estimated from the field difference between the two outer lines in the TREPR spectra. The spectra show that *D* is 12.5 and 40 mT for the quintet and triplet state spectra, respectively. The difference between the features observed in the TREPR spectra is very close to the expected difference between the quintet and triplet sublevels for a coupled triplet pair in the strong exchange limit, where the corresponding quintet state should exhibit a splitting of *D*/3, if the triplet state has a ZFS of *D* (ref. 31).

To assign the spin multiplicity, *S*, of the TREPR spectra definitively, electron spin echo-detected transient nutation experiments were performed. Upon application of a microwave field, *B*_1_, perpendicular to the external magnetic field, *B*_0_, a spin will begin nutating around the *B*_1_ vector with a frequency *ω*_nut_. The nutation frequency relates to the microwave field strength^32^ by:


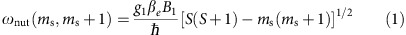


where *m*_s_ denotes the electron spin magnetic quantum number and *g*_1_ is the *g*-tensor referenced to the same axis as *B*_1_. For a spin ½ doublet state, equation (1) simplifies to:


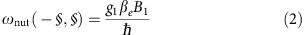


whereas for a triplet state:


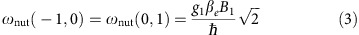


or for the two transitions from the *m*_s_=0 sublevel of a quintet state:


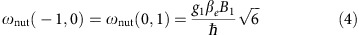


Figure 5b shows the spin nutation spectra obtained for NC at 344.5 and 358 mT, 600 ns and 15 μs after photoexcitation. Please note that the frequency axes of the nutation spectra were normalized to the nutation frequency of an *S*=½, α,γ-bisdiphenylene-β-phenylallyl (BDPA), free radical doublet state standard collected under the same experimental conditions. At 600 ns and 15 μs, the transitions at 344.5 mT show a *ω*_nut_/*ω*_BDPA_ of 2.44 for NC. These values are within experimental error of those expected for a quintet state. The transitions at 358 mT for NC show *ω*_nut_/*ω*_BDPA_ values of 1.5 and 1.44 at 600 ns and 15 μs, respectively, within experimental error of those expected for a triplet excited state. Therefore, the spin nutation experiments show conclusively that the narrow spectrum observed at early times in NC is that of a quintet state, while the broader feature observed prominently at later times is that of a triplet state.

### Theory and computational studies

To investigate the mechanism of SF and to reveal the role of |^5^(T_1_T_1_)〉, we have characterized the most relevant electronic states involved in the process in both the adiabatic and diabatic representations for the most stable conformation of NC (Supplementary Tables 1 and 4) using multireference perturbation theory calculations. The *i*-Bu_3_Si-groups were modelled using *i*-Pr_3_Si to avoid problems with conformational sampling. The results obtained show that the adiabatic lowest-lying singlet excited state S_1_ is of multiexcitonic character, that is, featuring large |^1^(T_1_T_1_)〉 contributions. This state is 0.04 eV more stable than the absorbing states, S_2_ and S_3_, which are essentially local excitations seen in pentacene monomers. They exhibit, in good agreement with experiments, vertical excitation energies of ∼1.94 eV (Supplementary Figs 8 and 9). Two dark CT states, S_5_ and S_6_, in which a charge is transferred from one pentacene to the other, lay ∼0.4 eV higher in energy than S_2_ and S_3_.

Using Truhlar’s fourfold-way diabatization procedure^33^, we have obtained the diabatic electronic states and the electronic couplings relevant for the characterization of the mechanism of the first step of SF (Supplementary Methods 4). These are the singlet ground state |S_0_S_0_〉, the correlated triplet pair (or multi-exciton) state |^1^(T_1_T_1_)〉, the locally excited singlet states |^1^(S_1_S_0_)〉 and |^1^(S_0_S_1_)〉 and the CT states |^1^(CA)〉 and |^1^(AC)〉 (refs 2, 3, 26, 34). To assess the relevance of the different SF mechanisms, we have calculated the effective coupling, *V*_eff_, of |^1^(S_1_S_0_)〉 to |^1^(T_1_T_1_)〉 (or |^1^(S_0_S_1_)〉 to |^1^(T_1_T_1_)〉)^35^,^36^:


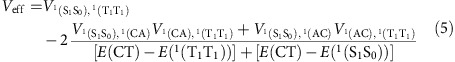


In this expression, *E*(*i*) are the energies of the corresponding diabatic states and *V*_*i*,*j*_ are the couplings between the states *i* and *j*. It is assumed that the mixing of the CT states with |^1^(T_1_T_1_)〉 and |^1^(S_1_S_0_)〉(|^1^(S_0_S_1_)〉) can be described perturbatively. This expression allows dissecting the contributions of the direct and mediated (via CT states) SF channels, represented by the first and second term on the right hand side of equation (5), respectively. Our results (Supplementary Table 4) show that while |^1^(T_1_T_1_)〉 couples significantly to |^1^(CA)〉 and |^1^(AC)〉 (101 and 99 meV, respectively), couplings to |^1^(S_1_S_0_)〉 and |^1^(S_0_S_1_)〉 are an order of magnitude smaller (5 and −11 meV, respectively). The calculated *V*_eff_ values for both |^1^(S_1_S_0_)〉 and |^1^(S_0_S_1_)〉 (Supplementary Table 6) show that the two states couple similarly to |^1^(T_1_T_1_)〉, but differ in the relative contributions based on the direct and mediated terms. For |^1^(S_1_S_0_)〉, the effective coupling is dominated by the mediated term, while for |^1^(S_0_S_1_)〉 the direct term is most important. Taking the SF quantum yields into account, these results suggest a mediated superexchange-like mechanism involving high-lying CT states. This has been corroborated using semiempirical (AM1) configuration-interaction (full CI) calculations with 8 (4+4) active orbitals and using a polarized continuum model with benzonitrile as model solvent^37^,^38^,^39^. The results show a 0.7 eV preferential energy lowering for the |^1^(CA)〉, |^1^(AC)〉, |^3^(CA)〉 and |^3^(AC)〉 states. This brings the CT states within 0.6 eV of |^1^(T_1_T_1_)〉 and |^5^(T_1_T_1_)〉, rendering the superexchange channel even more likely.

Finally, the experimental data show the involvement of a quintet spin triplet pair in the mechanism of SF as a precursor to the formation of the uncorrelated pair of triplet excited states. To substantiate this hypothesis further, we have calculated the energies of the lowest-lying quintet and triplet states of NC (Supplementary Table 1). The results show that the lowest-lying quintet state |^5^(T_1_T_1_)〉 is nearly degenerate with the two lowest-lying bright states and with |^1^(T_1_T_1_)〉. Therefore, population of |^5^(T_1_T_1_)〉 through spin-mixing is possible (Supplementary Methods 4)^2^,^8^, eventually leading to the formation of the uncorrelated triplet pair. The calculated state energies are fully compatible with the kinetic model in Fig. 3. Further computational modelling will be required to understand more fully the role of spin–spin interactions and electronic coupling between the relevant states in the SF mechanism.

## Discussion

The energy splitting between the ^1^(T_1_T_1_) and ^5^(T_1_T_1_) states depends on the exchange interaction (*J*_SQ_) between them. If two pentacenes interact strongly in a π-stacked geometry, as may occur in a polycrystalline solid, *J*_SQ_ is large, so that the initially formed ^1^(T_1_T_1_) does not mix with ^5^(T_1_T_1_). If the rate at which the two triplets within ^1^(T_1_T_1_) diffuse apart in the material is faster than singlet-quintet spin-mixing, a process that usually takes place in nanoseconds, then the direct formation of (T_1_+T_1_) from ^1^(T_1_T_1_) may occur. However, if the triplet excitons separate on a timescale comparable to spin-mixing, the exponential decrease in *J*_SQ_ as the distance between the two spin-correlated triplet states increases will result in singlet-quintet mixing^40^,^41^. In contrast, if two pentacenes are covalently linked, *J*_SQ_ is controlled by the electronic structure of the linkage between them. Thus, the non-conjugated spacer in NC ensures that *J*_SQ_ is small enough that singlet-quintet spin-mixing occurs on a timescale that can be directly observed by TREPR. Subsequent formation of two independent triplet states, which each carry spin polarization indicative of the quintet state from which they are derived, is followed by spin relaxation and subsequent triplet decay to the ground state.

The value of *J*_SQ_ is independent of the magnetic field; however, the ISC rates between ^1^(T_1_T_1_) and ^5^(T_1_T_1_) may depend on the magnetic field because the field causes Zeeman splitting of the magnetic sublevels of ^5^(T_1_T_1_). The TREPR data show that ISC between ^1^(T_1_T_1_) and ^5^(T_1_T_1_) occurs primarily via the *m*_s_=0 sublevel in the presence of a magnetic field. Since the *m*_s_=0 sublevel energy is field invariant, this implies that at the ∼350 mT applied field, the value of *J*_SQ_ is large enough that the *m*_s_=±1 and *m*_s_=±2 sublevels of ^5^(T_1_T_1_) are sufficiently far removed energetically from ^1^(T_1_T_1_) to prevent significant mixing. In contrast, in the absence of a magnetic field with no Zeeman splitting, the magnetic sublevels of ^5^(T_1_T_1_) are nearly degenerate and are split only by the weak dipolar interaction between the two adjacent triplet states. Thus, mixing between ^1^(T_1_T_1_) and three of the five ^5^(T_1_T_1_) sublevels having appropriate symmetry^42^ should increase the ISC rates between them, which may account for the 2–3 fold increase in the ISC rates *k*_3_ and *k*_−3_ going from 105 to 295 K (Table 1).

In summary, linking two pentacenes using a non-conjugated 1,3-diethynyladamantyl spacer results in sufficient electronic coupling between the pentacenes to render SF facile, while at the same time keeping the coupling sufficiently weak to allow spin decoherence leading to two independent triplet states to compete successfully with triplet–triplet annihilation leading back to the ^1^(S_1_S_0_) state. In a highly viscous butyronitrile matrix at cryogenic temperatures, we directly observe spin-mixing of ^1^(T_1_T_1_) with ^5^(T_1_T_1_) followed by spin decoherence yielding separated (T_1_+T_1_) states. The combination of transient optical and electron paramagnetic resonance (EPR) data provides a means of identifying and analysing the SF mechanism over a very broad time range, which should prove very useful in optimizing molecular designs for SF.

## Methods

### Spectroscopy

Anthracene and benzonitrile were purchased from commercial suppliers and used without further purification. Butyronitrile (PrCN) was purchased from Alfa-Aesar and treated with HCl, distilled from K_2_CO_3_ and dried over Al_2_O_3_ (ref. 43).

Steady-state ultraviolet–visible absorption spectra were acquired at 295 K using a PerkinElmer Lambda 2 spectrometer and at 105 K using a Shimadzu UV-1800. Steady-state fluorescence spectra were measured with a Horiba FluoroMax3 spectrometer in the visible detection range.

fsTA and nsTA experiments (295 K) were carried out with an amplified Ti:Sapphire CPA-2110 fs laser system (Clark MXR: output 775 nm, 1 kHz, 150 fs pulse width) using transient absorption pump/probe detection systems (Helios and Eos, Ultrafast Systems) with argon-purged solutions. The 610 and 656 nm excitation wavelengths were generated with a noncollinear optical parametric amplifier (NOPA, Clark MXR). The 387 nm excitation wavelength was generated via second harmonic generation (SHG) of the 775 nm laser output.

fsTA and nsTA experiments (105 K) were carried out with an amplified Ti:Sapphire Spitfire Pro fs laser system (Spectra-Physics: output 827 nm, 1 kHz, 100 fs pulse width) using transient absorption pump/probe detection systems (customized Helios and Eos, Ultrafast Systems) in a liquid nitrogen cryostat (VNF-100, Janus Research Company). The 590 and 610 nm excitation wavelengths were generated with a laboratory-constructed collinear optical parametric amplifier^44^.

Samples for fsTA and nsTA at 105 K were prepared as dichloromethane solutions with an optical density between 0.1 and 0.3 at 590 and 610 nm in 2 mm quartz cuvettes, respectively. The dichloromethane was evaporated and the samples reconstituted with an equal volume of PrCN in an N_2_-filled glove box. The resulting solutions were loaded into an optical sample cell consisting of two quartz windows separated by a 2 mm PTFE spacer, and the cell was sealed under N_2_. Low-temperature transient absorption measurements were performed using a Janis VNF-100 cryostat and a Cryo-Con 32B temperature controller. Low-temperature samples in PrCN were cooled rapidly to 77 K by flooding the cryostat with liquid N_2_ to produce a cracked glass. Samples were then annealed at 120 K to remove cracks and cooled to 105 K. At 105 K, PrCN is a viscous, supercooled liquid with a dielectric relaxation time of ∼5 ms (ref. 45). Since the timescale of solvent motion at this temperature is much longer than that of the photophysics observed here, PrCN at 105 K can be treated as a solid for the purposes of this study.

Global analysis was performed on the transient absorption data sets using an in-house written program in Matlab (The Mathworks, Inc.) previously described^46^. The analytic solution to the coupled differential equations that describe the kinetic model is convoluted with a Gaussian instrument response function. After the least-squares fitting has converged, the raw data matrix is deconvoluted using the specific solution to the kinetic model and parameters from the fit to obtain the species-associated spectra and their populations as a function of time.

EPR measurements were made at the X-band (∼9.5 GHz) using a Bruker Elexsys E680-X/W EPR spectrometer outfitted with a split ring resonator (ER4118X-MS5). Measurements were performed at 105 and 5 K using an Oxford Instruments CF935 continuous flow cryostat with liquid N_2_ or He, respectively. TREPR measurements were performed following photoexcitation with 7 ns, 2.5 mJ, 640 nm pulses using the output of an optical parametric oscillator (Spectra-Physics Basi-scan), pumped with the output of a frequency tripled Nd:YAG laser (Spectra-Physics Quanta-Ray Pro 350).

Transient EPR spectra were collected following laser photoexcitation, the transient magnetization was detected in quadrature under continuous wave microwave irradiation (5 mW), without field modulation. Sweeping the magnetic field gave two-dimensional spectra versus both time and magnetic field. For each kinetic trace, the signal acquired prior to the laser pulse was subtracted from the data. Kinetic traces recorded at magnetic field values off resonance were considered background signals, whose average was subtracted from all kinetic traces.

Pulsed experiments employed a 1 kW travelling wave tube (TWT) amplifier (Applied Systems Engineering 117X) to generate high-power microwave pulses. The resonator was partially overcoupled to maximize echo intensity and minimize ringing following microwave pulses. The spin nutation experiment was performed with a *P*_nut_−*T*−*π*/2−*τ*−*π* pulse sequence; the nutation pulse, *P*_nut_, was of variable length, the delays were *T*=600 and *τ*=200 ns, the *π*/2 and *π* denote microwave pulses of lengths 16 and 32 ns, respectively, and were optimized to provide ideal turning angles for the quintet spin echo. The samples were photoexcited 600 ns and 15 μs prior to the microwave pulse sequence. To avoid spin relaxation effects, the pulsed experiments were performed at 5 K. At 105 K, the temperature at which the transient continuous wave (TCW) EPR was acquired, the spin echo for the triplet signal was not clearly resolved; however, significant echo intensity was observed at 5 K.

### Computational details

The ground-state equilibrium geometries were optimized using density functional theory, employing the B3LYP correlation-exchange functional^47^ and Pople’s 6-31G(d) basis set^48^,^49^,^50^. Dispersion interactions were incorporated using Grimme’s empirical dispersion correction^51^. Vertical excitation energies were calculated using the extended multi-configurational quasi-degenerate perturbation theory^52^ method using a double-ζ basis set^53^, employing state average complete active space self-consistent field (SA-CASSCF)^54^ wave-functions as reference and an intruder state avoidance shift of 0.02 a.u. (ref. 55). The active space employed in the SA-CASSCF calculations comprised eight electrons in eight orbitals (highest occupied molecular orbital (HOMO)−1, HOMO, lowest unoccupied molecular orbital (LUMO) and LUMO+1 per pentacene unit, see Supplementary Methods 4) and 15 roots with equal weights were used. Dipole moments and oscillator strengths were calculated at the SA-CASSCF level. Diabatic states were built using Truhlar’s fourfold-way diabatization method^33^,^56^. In the diabatic procedure, we used the eight lowest-lying adiabatic singlet electronic states obtained with an eight roots equal weights SA-CASSCF calculation. In these calculations, an active space of four electrons in four orbitals (HOMO and LUMO per pentacene unit) was used. All calculations were carried out using the electronic structure calculation packages GAUSSIAN09 (ref. 57) and GAMESS^58^,^59^.

### Data availability

The data that support the findings of this study are available from the corresponding authors on request.

## Additional information

**How to cite this article:** Basel, B. S. *et al*. Unified model for singlet fission within a non-conjugated covalent pentacene dimer. *Nat. Commun.* **8,** 15171 doi: 10.1038/ncomms15171 (2017).

**Publisher’s note:** Springer Nature remains neutral with regard to jurisdictional claims in published maps and institutional affiliations.

## Supplementary Material

Supplementary Information Supplementary Figures, Supplementary Methods, Supplementary Tables and Supplementary References

## Figures and Tables

**Figure 1:**
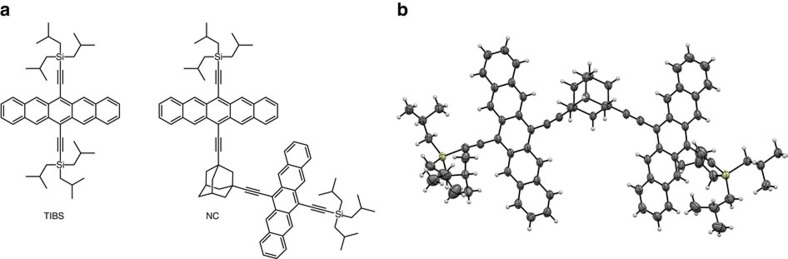
Molecular and solid-state structures. (**a**) Chemical structures of the pentacene monomer (TIBS) and dimer (NC). (**b**) X-ray crystallographic structure of NC illustrating the arrangement and proximity of the two pentacenes.

**Table 1:** Rate constants for NC using the kinetic model shown in Fig. 3.

	***k***_**1**_ **(s**^**−1**^**)**	***k***_**2**_ **(s**^**−1**^**)**	***k***_**−2**_ **(s**^**−1**^**)**	***k***_**3**_ **(s**^**−1**^**)**
295 K	9.5±0.7 × 10^7^	2.4±0.1 × 10^9^	5.0±0.3 × 10^7^	1.1±0.1 × 10^7^
105 K	5.1±0.2 × 10^7^	3.3±0.9 × 10^8^	1.0±0.1 × 10^6^	6.7±0.1 × 10^6^
				
	***k***_**−3**_ **(s**^**−1**^**)**	***k***_**4**_ **(s**^**−1**^**)**	***k***_**5**_ **(s**^**−1**^**)**	***k***_**6**_ **(s**^**−1**^**)**
295 K	4.8±0.1 × 10^6^	1.0±0.1 × 10^7^	5.7±0.1 × 10^6^	3.1±0.1 × 10^4^
105 K	1.8±0.1 × 10^6^	4.1±0.1 × 10^6^	6.7±0.1 × 10^5^	2.5±0.1 × 10^4^
