# Supplementary material for: Unified model for singlet fission within a non-conjugated covalent pentacene dimer
Source: Nat Commun. 2017 May 18;8:15171. doi: 10.1038/ncomms15171 (PMC5493541; doi:10.1038/ncomms15171)
Supplement: Supplementary Information — Supplementary Figures, Supplementary Methods, Supplementary Tables and Supplementary References [file ncomms15171-s1.pdf]

Supplementary Methods 1 | Synthesis and Characterization.

Reagents were purchased reagent grade from commercial suppliers and used without further purification. α,γ-Bisdiphenylene-β-phenylallyl (BDPA) was purchased from Sigma-Aldrich. Compound **S1** and 6,13-bis(tri-*iso*butylsilylethynyl)pentacene (**TIBS**) were synthesized as reported.[1, 2] THF was distilled from Na/benzophenone ketyl. All reactions were performed in standard dry glassware. $MgSO_4$ was used as the drying agent after aqueous workup. Evaporation and concentration *in vacuo* was done at water-aspirator pressure. Brine refers to a saturated aqueous solution of NaCl.

^1H and ^{13}C spectra were recorded on a Bruker Avance instrument at 400 MHz (^1H) and 100 MHz (^{13}C). NMR spectra were referenced to the residual solvent signal (1H: $CDCl_3$, 7.24 ppm; ^{13}C: $CDCl_3$, 77.0 ppm) and recorded at ambient temperature. $CDCl_3$ (99.8%, Deutero GmbH) was stored over 4 Å molecular sieves. Coupling constants are reported as observed (±0.5 Hz). UV-vis measurements were carried out on a Varian Cary 5000 UV-vis-NIR spectrophotometer at rt. Mass spectra were obtained from Bruker micro TOF II or Bruker maxis 4G (APPI, ESI) instruments. IR spectra were recorded as solids on Varian 660-IR or Bruker Tensor 27-IR spectrometers in ATR-mode. Melting points were measured with an Electrothermal 9100 instrument. TLC analyses were carried out on TLC plates from Marchery-Nagel (Alugram® SIL G/UV_{254}) and visualized via UV-light (264/364 nm) or standard coloring reagents. Column chromatography was performed using Silica Gel 60M (Merck). Differential scanning calorimetry (DSC) measurements were made on a Mettler Toledo TGA/ STDA 851e/1100/SF. X-ray data for **NC** (CCDC 1503240) has been deposited at the Cambridge Crystallographic Data Centre (CCDC), 12 Union Road, Cambridge CB21EZ, UK; fax: (+44)122-333-6033. These data can be

obtained free of charge from The Cambridge Crystallographic Data Centre via the Internet at www.ccdc.cam.ac.uk/data_request/cif using the CCDC numbers given above.

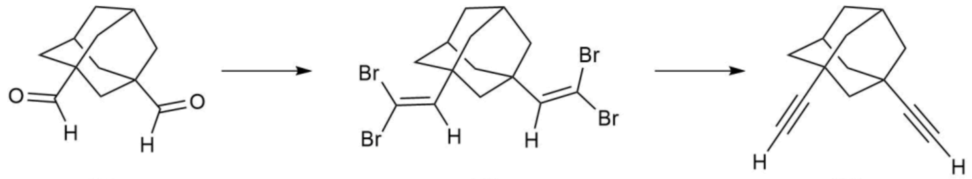

S1 S2 S3

Supplementary Figure 1 | Chemical Structures of S1, S2 and S3.

Supplementary Figure 1, S3. At 0 °C, CBr_4 (5.61 g, 16.9 mmol) was dissolved in dry, deoxygenated CH_2Cl_2 (100 mL), and PPh_3 (9.27 g, 35.3 mmol) was added within 15 min as a solid. The mixture was allowed to warm to room temperature and stirred for further 30 min. A solution of **Supplementary Figure 1, S1** (0.700 g, 3.64 mmol) in dry, deoxygenated CH_2Cl_2 (20 mL) was added dropwise *via* cannula within 15 min and the mixture was allowed to stir for 18 h. Hexanes (200 mL) was added and the resulting suspension was filtered through a pad of silica (hexanes), washed with hexanes (3 × 50 mL), and the solvent removed *in vacuo*. The resulting solid, **Supplementary Figure 1, S2**, was directly subjected to elimination without further purification.

At –78 °C, the crude dibromoolefin **Supplementary Figure 1, S2** (1.10 g, 2.20 mmol) was dissolved in dry, deoxygenated THF (30 mL) and *n*-BuLi (3.70 mL, 9.25 mmol, 2.5 M in hexanes) was added dropwise *via* cannula within 10 min. The mixture was stirred at –78 °C for 1 h and then for 1.5 h at room temperature. Satd. aq. NH_4Cl (100 mL) was added and the aqueous phase was extracted with CH_2Cl_2 (3 × 50 mL). The combined organic phases were washed with water (100 mL), brine (100 mL), dried ($MgSO_4$), and the solvent removed *in vacuo*. Column chromatography (silica, CH_2Cl_2/hexanes 1:1) afforded **Supplementary Figure 1, S3** (0.349 g,

52% based on **S1**) as a colorless solid. Physical and spectroscopic data were consistent with those reported.[3, 4]

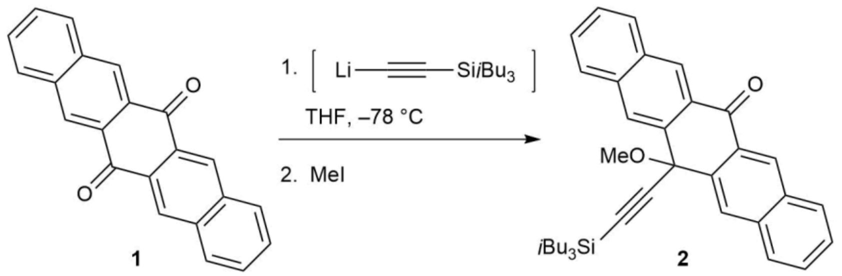

Supplementary Figure 2 | Chemical Structures of 1 and 2.

Supplementary Figure 2, 2. At –78 °C, tri-*iso*butylsilylacetylene (1.79 g, 8.00 mmol) was dissolved in dry, deoxygenated THF (10 mL), and *n*-BuLi (3.20 mL, 8.00 mmol; 2.5 M in hexanes) was added *via* cannula. The mixture was stirred at –78 °C for 15 min. This solution was transferred *via* cannula to a suspension of **Supplementary Figure 2, 1** (2.50 g, 8.11 mmol) in dry, deoxygenated THF (40 mL) at room temperature and stirred for 5 h. MeI (5.70 g, 2.50 mL, 40.2 mmol) was added, and the mixture was stirred for 18 h. Satd. aq. NH_4Cl (100 mL) was added and the aqueous phase was extracted with CH_2Cl_2 (3 × 50 mL). The combined organic phases were washed with water (100 mL), brine (100 mL), dried ($MgSO_4$), and the solvent removed *in vacuo*. The residue was dissolved in CH_2Cl_2 (5 mL), MeOH (70 mL) was added, and the mixture was cooled to –15 °C. The resulting suspension was filtered, and the residue was washed with MeOH (3 × 10 mL) affording **Supplementary Figure 2, 2.** (3.28 g, 75%) as a yellow solid. Mp 110 – 112 °C. R_f = 0.89 (CH_2Cl_2). IR (ATR, solid state) 3058 (w), 2949 (m), 2864 (m), 1670 (m) cm^{-1}. ^1H NMR (400 MHz, $CDCl_3$) δ 8.82 (s, 2H), 8.62 (s, 2H), 8.05 (d, J = 8.0 Hz, 2H), 7.94 (d, J = 8.0 Hz, 2H), 7.65 (t, J = 7.0 Hz, 2H), 7.58 (t, J = 7.3 Hz, 2H), 3.10 (s, 3H), 2.06 (nonet, J = 6.7 Hz, 3H), 1.10 (d, J = 6.6 Hz, 18H), 0.85 (d, J = 7.0 Hz, 6H). ^{13}C

NMR (100 MHz, $CDCl_3$) δ 185.0, 135.8, 135.0, 132.9, 129.8, 129.5, 128.8, 128.22, 128.16, 127.4, 103.8, 95.6, 75.0, 52.2, 26.4, 25.2, 25.1 (one signal coincident or not observed). LDI MS *m/z* 515 ($[M – OMe]^+$, 100). APPI HRMS *m/z* calcd. for $C_{37}H_{42}O_2Si$ (M^+) 546.2949, found 546.2938, calcd. for $C_{36}H_{39}OSi$ ($[M – OMe]^+$) 515.2765, found 515.2766.

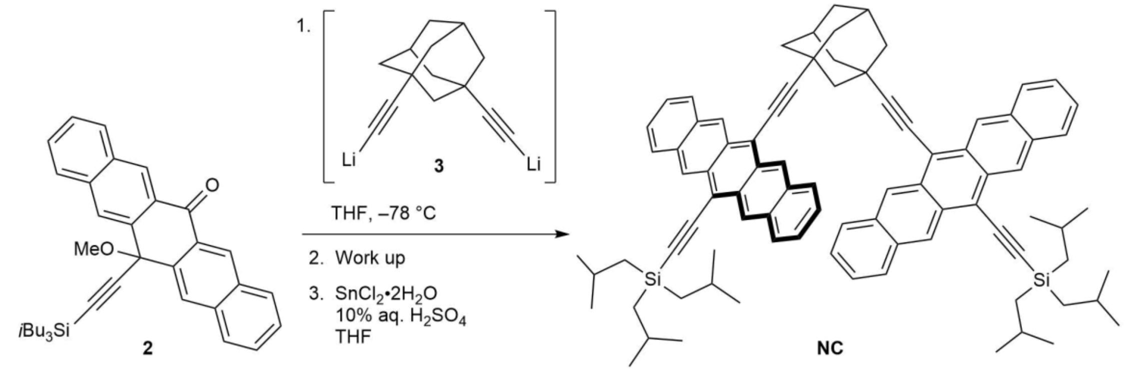

Supplementary Figure 3 | Chemical Structures of 3 and NC.

Supplementary Figure 3, NC. To a solution of **Supplementary Figure 1, S3** (0.150 g, 0.815 mmol) in dry, deoxygenated THF (20 mL) at –78 °C was added LiHMDS (1.90 mL, 1.90 mmol, 1 M in THF/ethylbenzene) and the mixture was stirred for 45 min at –78 °C, giving a solution of **Supplementary Figure 3, 3.** A solution of **Supplementary Figure 2, 2** (0.923 g, 1.69 mmol) in dry, deoxygenated THF (15 mL) was added *via* cannula at –78 °C to the solution of **Supplementary Figure 3, 3** (as prepared above), and the resulting mixture was allowed to warm to room temperature and stirred for further 18 h. Satd. aq. NH_4Cl (100 mL) was added, and the aqueous phase extracted with CH_2Cl_2 (3 × 50 mL). The combined organic phases were washed with water (100 mL), brine (100 mL), dried ($MgSO_4$), and the solvent removed *in vacuo*. The band that corresponded to a R_f value of 0.70 was collected *via* column chromatography (silica, CH_2Cl_2) and subjected to reductive aromatization without further purification. To a solution of the crude intermediate (0.336 g, 0.263 mmol) in dry, deoxygenated THF (15 mL) was added $SnCl_2•2H_2O$ (0.593 g, 2.63 mmol) and 10% H_2SO_4 (0.5 mL) at room temperature. The

flask was wrapped in aluminum foil in order to limit light exposure. The mixture was stirred for 5 h and then poured into MeOH (70 mL) and cooled to –78 °C. The suspension was filtered, and the residue was washed with MeOH (3 × 20 mL). The solid was dissolved in CH_2Cl_2 (30 mL), washed with water (100 mL), brine (100 mL), dried ($MgSO_4$), and the solvent removed by passing N_2 over the solution. Column chromatography (silica, CH_2Cl_2/hexanes 1:1) afforded **Supplementary Figure 3, NC** (0.385 g, 40% based on **S3**) as a deep blue solid. Mp 138 – 140 °C. R_f = 0.83 (CH_2Cl_2/hexanes 1:1). UV-vis (CH_2Cl_2) λ_{max} (ε): 272 (43200), 308 (377000), 326 (65700), 352 (15100), 438 (5840), 550 (8330), 591 (21100), 642 (39600). IR (ATR, solid state) 3048 (w), 2950 (m), 2922 (m), 2903 (m), 2865 (m), 2124 (m), 1461 (m) cm^{-1}.

^{1}H NMR (400 MHz, $CDCl_3$) δ 9.25 (s, 4H), 9.22 (s, 4H), 8.04 (d, J = 8.2 Hz, 4H), 7.94 (d, J = 8.1 Hz, 4H), 7.38–7.34 (m, 8H), 2.90 (s, 2H), 2.52–2.43 (m, 10H), 2.19 (nonet, J = 6.0 Hz, 6H), 2.01 (s, 2H), 1.19 (d, J = 6.6 Hz, 36H), 0.96 (d, J = 6.9 Hz, 12H). ^{13}C NMR (100 MHz, $CDCl_3$) δ 132.3, 132.1, 130.8, 130.2, 128.8, 128.5, 126.2, 126.1, 126.0, 125.8, 118.9, 117.5, 112.4, 109.3, 104.8, 78.4, 47.9, 42.3, 35.4, 31.9, 28.4, 26.6, 25.5 (one signal coincident or not observed). MALDI MS (dhb) *m/z* 1181 (M^+, 100). APPI HRMS calcd. for $C_{86}H_{92}Si_2$ (M^+) 1180.6732, found 1180.6729. DSC: decomposition, 282 °C (onset), 364 °C (peak). A crystal **NC** suitable for X-ray crystallography was grown by slow diffusion of MeOH into a solution of **NC** dissolved in a mixture of $CHCl_3$/benzene at 5 °C for several days. X-ray crystallographic data for **NC** ($C_{86}H_{92}Si_2$), F_w = 1181.77; triclinic crystal system, space group P-1; crystal size = 0.5 × 0.2 × 0.05 mm^3; a = 11.0469(3) Å, b = 14.7688(5) Å, c = 21.3858(5) Å; α = 103.024°, β = 94.353°, γ = 92.439°; V = 3383.10(17) Å^3; Z = 2; $\rho_{(calc)}$ = 1.160 g/cm^3; 2θ_{max} = 134.16°; μ = 0.812 mm^{-1}; T = 100.00(10) K; total data collected = 27627; R_1 = 0.0760 [9891 independent reflections with $I \geq 2\sigma(I)$]; ωR_2 = 0.2265 for 11974 data, 901 variables, and 140 restraints, largest difference, peak

and hole = 0.59 and –0.43 eÅ^{-3}. Disorder in atoms C60/61/62/61a/62a/62b/63/63a/63b were refined with fixed 33% occupation, C106/107/108:C10a/C10b/C10c with 66:34 occupation, and C110/111/112:C11a/C11b/C11c with 90:10 occupation. CCDC 1503240.

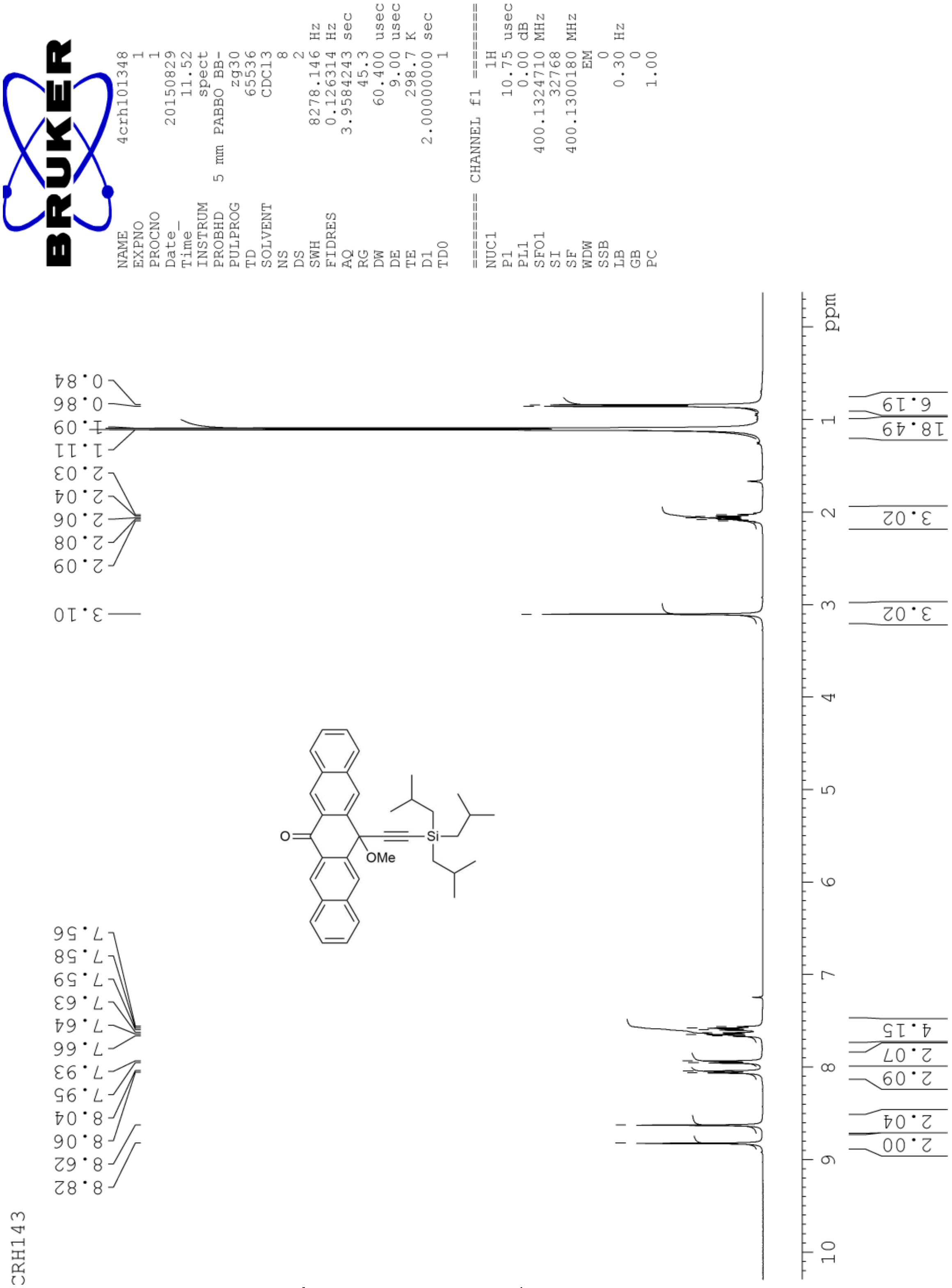

Supplementary Figure 4 | NMR spectrum. ^{1}H NMR spectrum of compound **2** (400 MHz, $CDCl_3$, rt).

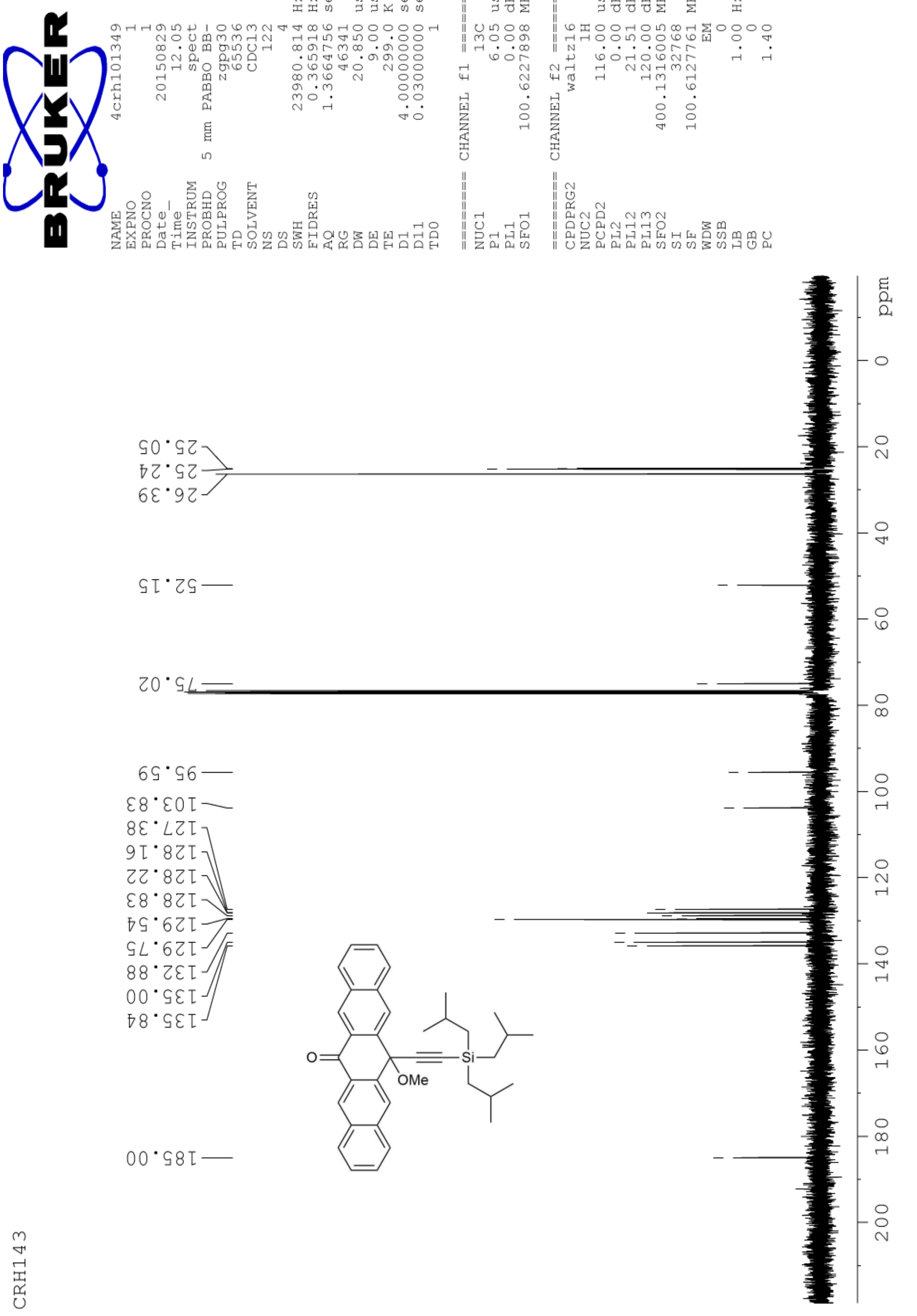

Supplementary Figure 5 | NMR spectrum. ^{13}C NMR spectrum of compound **2** (100 MHz, $CDCl_3$, rt).

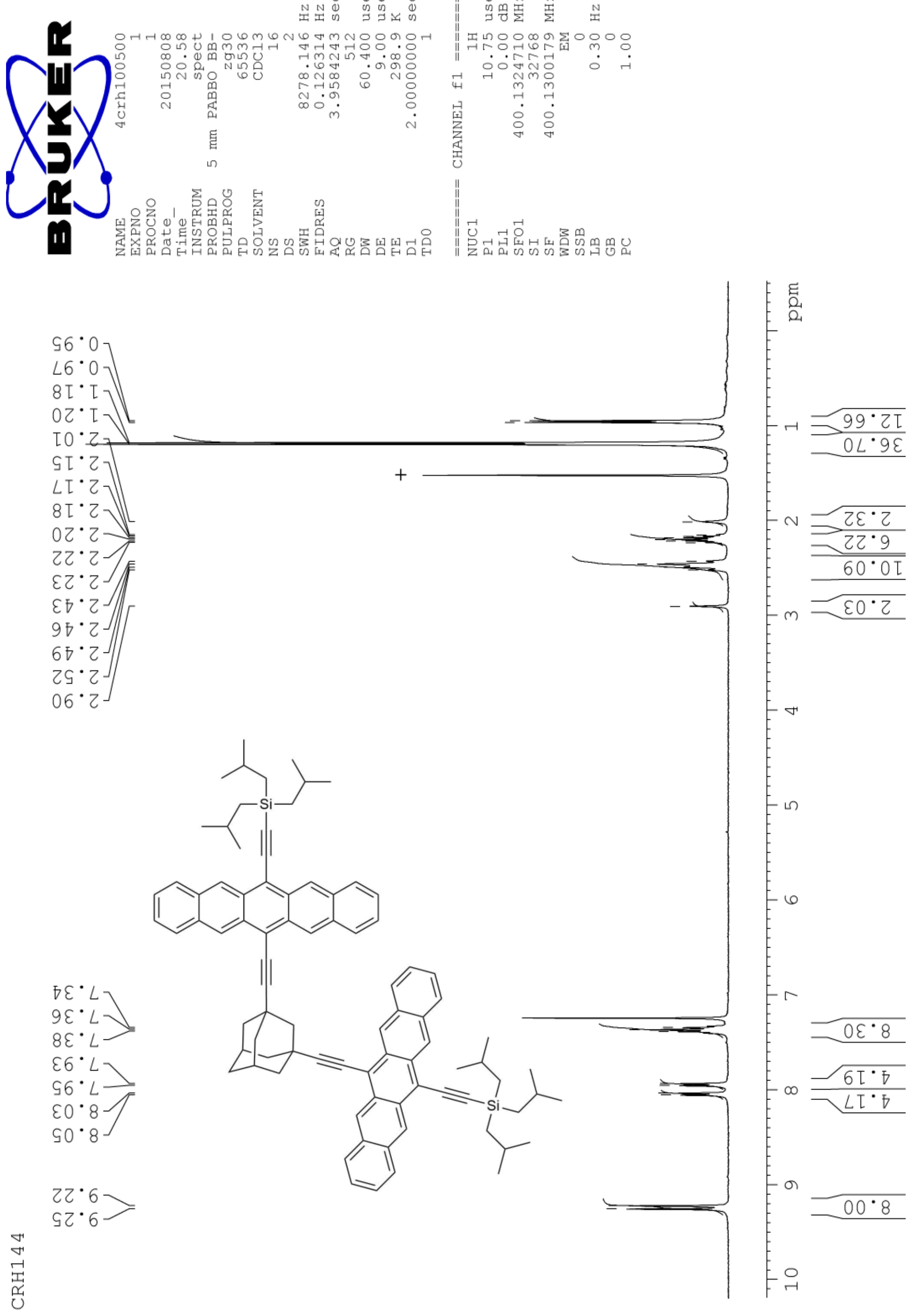

Supplementary Figure 6 | NMR spectrum. ^{1}H NMR spectrum of compound **NC** (400 MHz, $CDCl_3$, rt, + = H_2O).

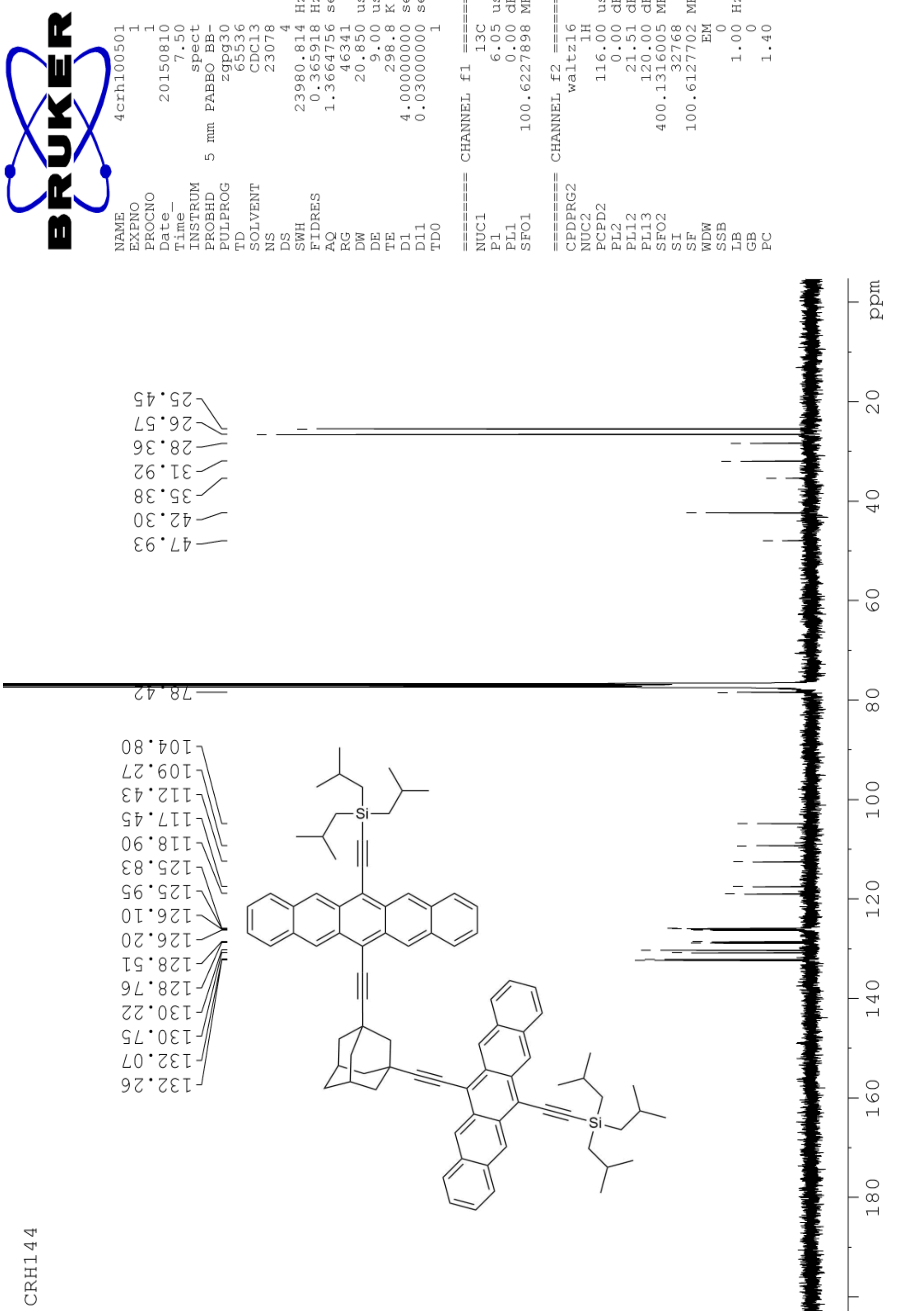

Supplementary Figure 7 | NMR spectrum. ^{13}C NMR spectrum of compound **NC** (100 MHz, $CDCl_3$, rt).

Supplementary Methods 2 | Photophysics.

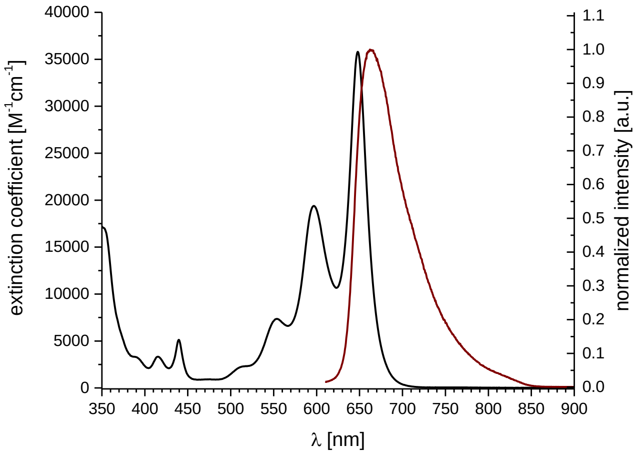

Supplementary Figure 8 | UV-Vis absorption and fluorescence spectra. Absorption spectrum of **NC** (black line) in benzonitrile solution at 295 K and normalized fluorescence spectrum of **NC** (brown line) in benzonitrile solution following photoexcitation at 590 nm. The corresponding fluorescence quantum yield in benzonitrile is 1.7% (measured relative to a zinc phthalocyanine used as a reference compound[5]).

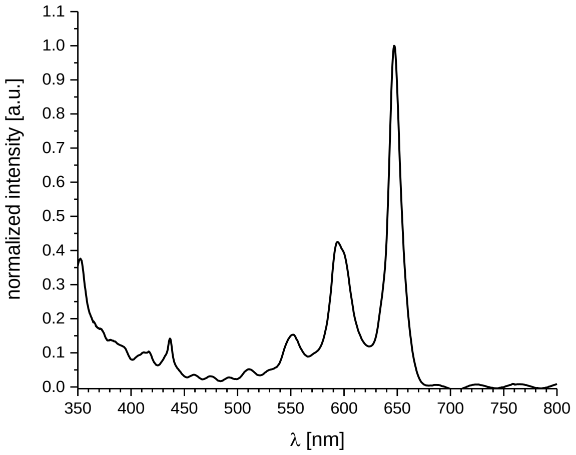

Supplementary Figure 9 | UV-Vis absorption spectrum. Absorption spectrum of **NC** at 105 K in butyronitrile.

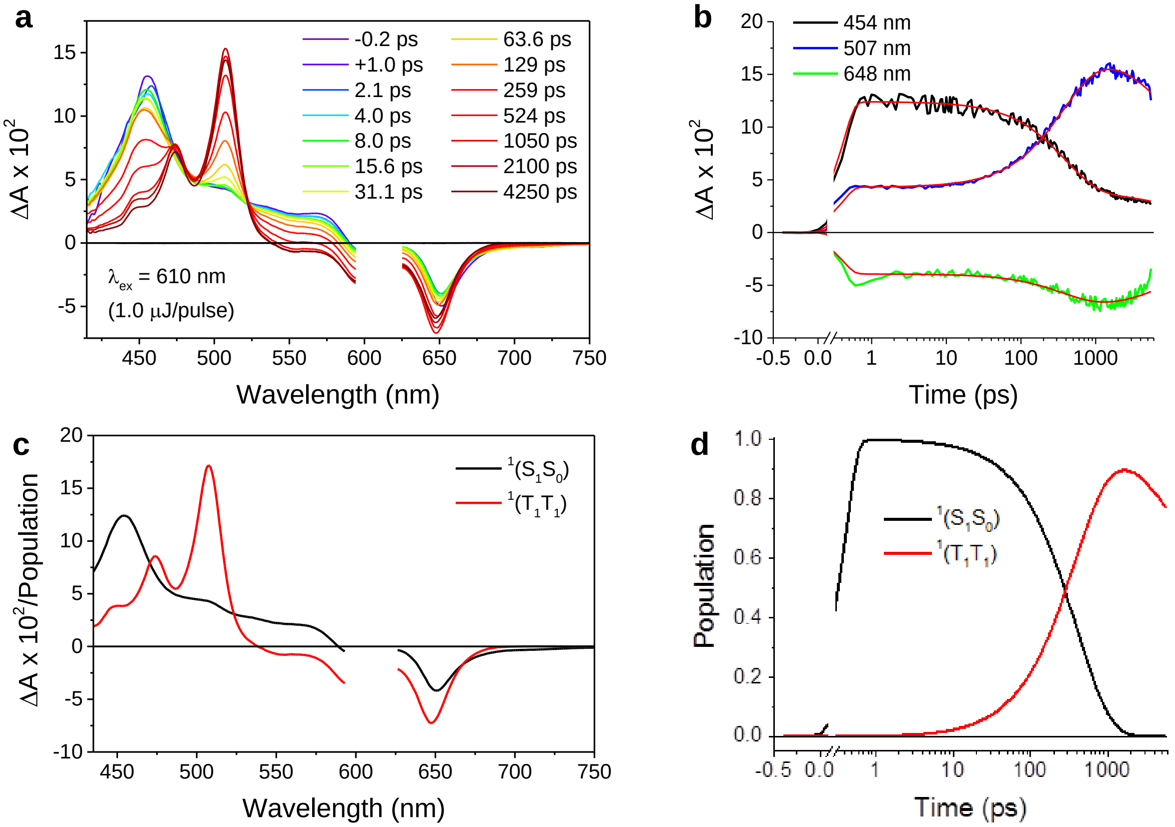

Supplementary Figure 10 | Transient absorption measurements. a) Transient absorption difference spectra for **NC** obtained at 295 K in benzonitrile. b) Single wavelength kinetics and fits to the data. c) Species-associated spectra using the kinetic model given in Fig. 3. d) Population kinetics for each species. The total number of T_1 states per S_1 created is twice the population of the T_1 species indicated in d). Multiplying the species-associated spectra in c) by the corresponding species populations in d) yields the complete ΔA vs. time and wavelength data set.

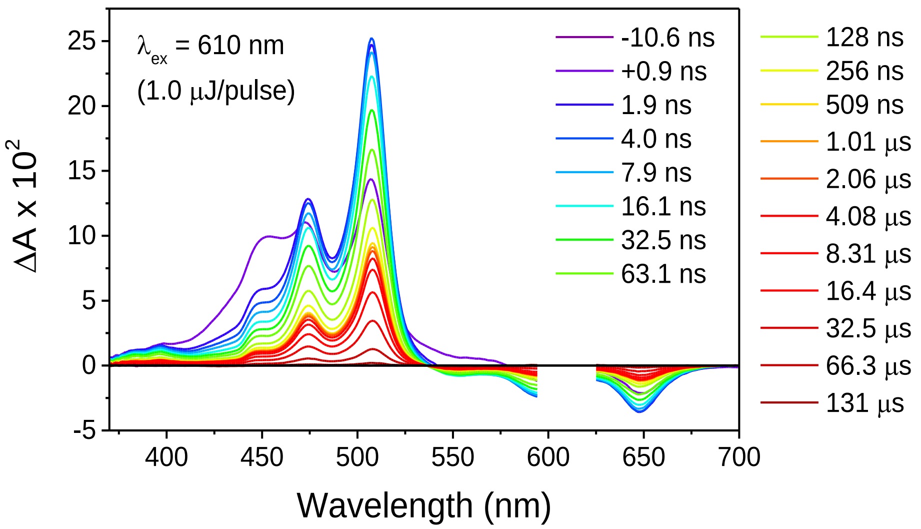

Supplementary Figure 11 | Transient absorption measurements. Transient absorption difference spectra for **NC** obtained at 295 K in benzonitrile at nanosecond and microsecond time scales.

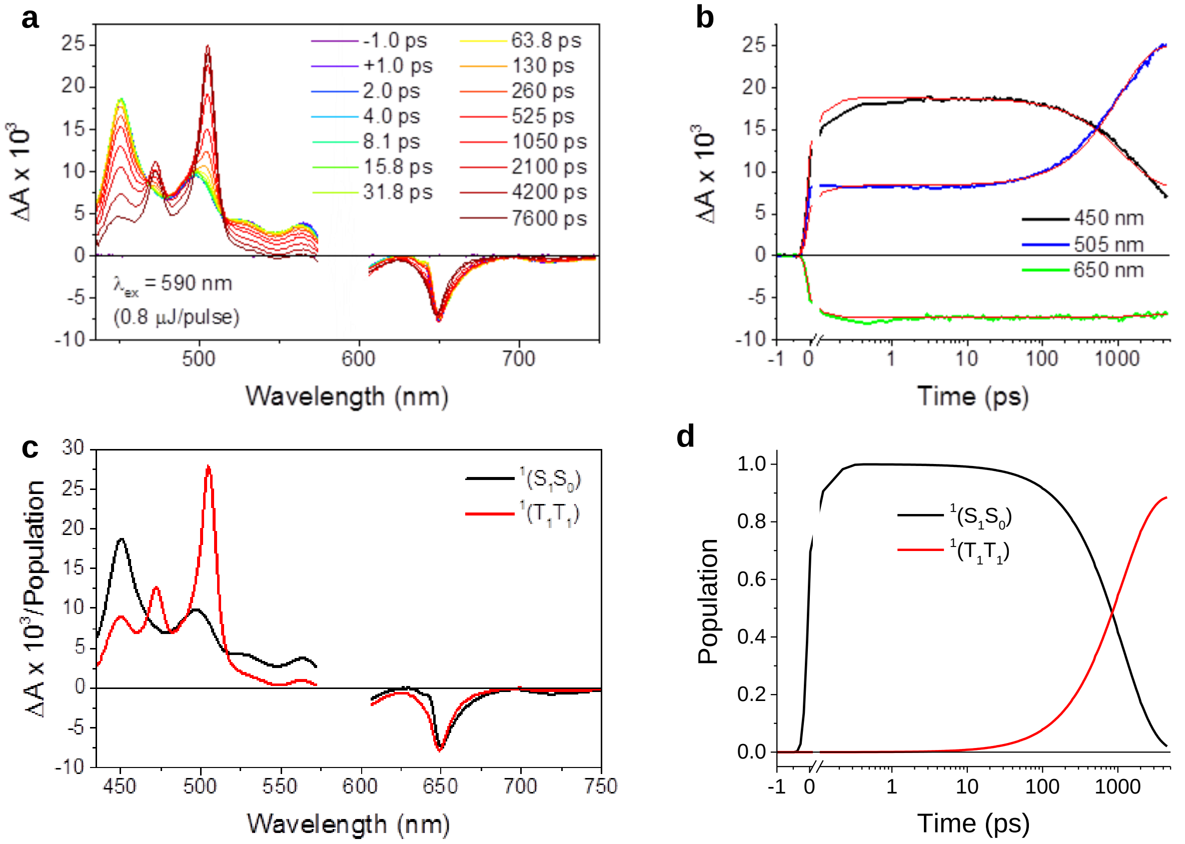

Supplementary Figure 12 | Transient absorption measurements. a) Transient absorption difference spectra for **NC** obtained at 105 K in butyronitrile. b) Single wavelength kinetics and fits to the data. c) Species-associated spectra using the kinetic model given in Fig. 3. d) Population kinetics for each species. The total number of T_1 states per S_1 created is twice the population of the T_1 species indicated in d). Multiplying the species-associated spectra in c) by the corresponding species populations in d) yields the complete ΔA vs. time and wavelength data set.

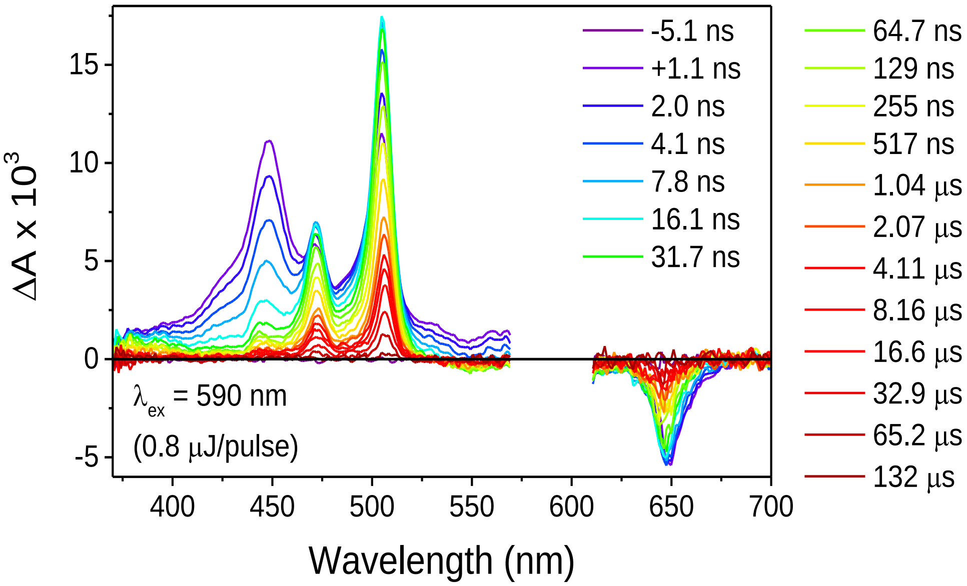

Supplementary Figure 13 | Transient absorption measurements. Transient absorption difference spectra for **NC** obtained at 105 K in butyronitrile at nanosecond and microsecond time scales.

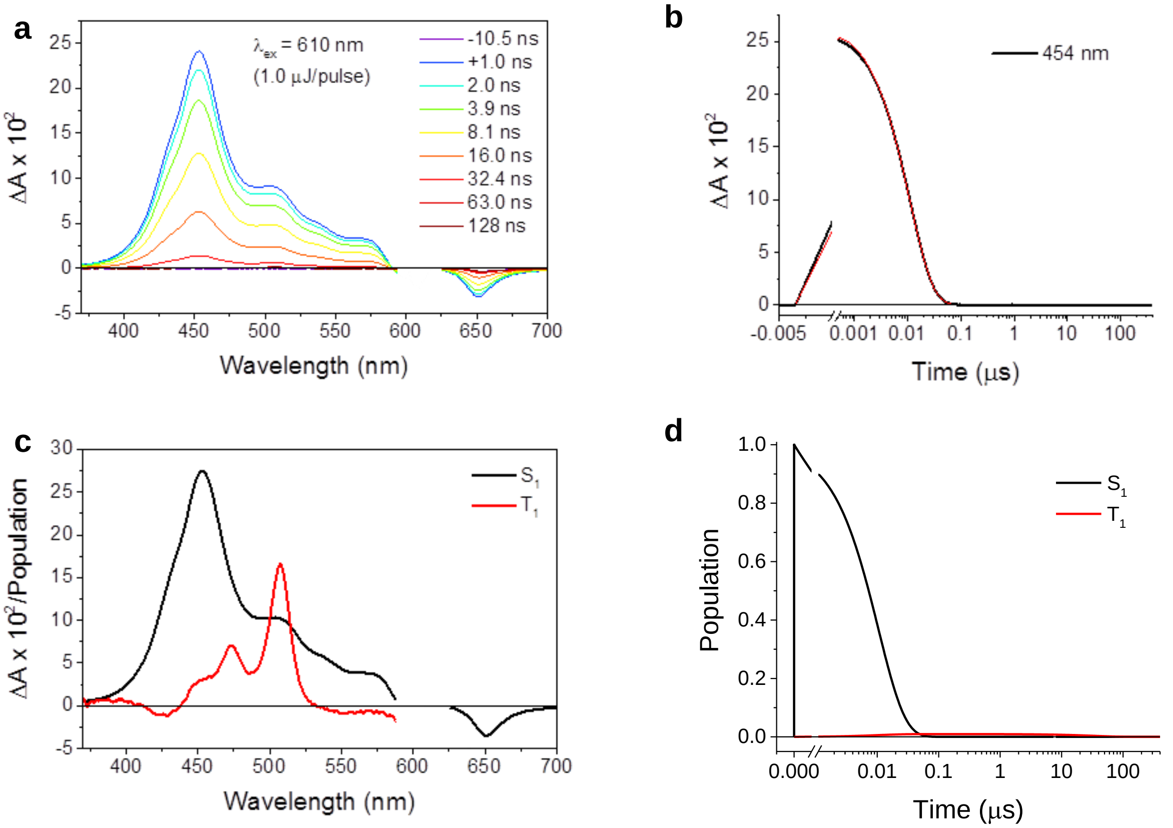

Supplementary Figure 14 | Transient absorption measurements. a) Transient absorption difference spectra for **TIBS** obtained at 295 K in benzonitrile. b) Single wavelength kinetics and fits to the data. c) Species-associated spectra using the kinetic model given in Fig. 3. d) Population kinetics for each species. Multiplying the species-associated spectra in c) by the corresponding species populations in d) yields the complete ΔA vs. time and wavelength data set. The data yield the decay constants for S_1: $k_{S1} = 9.5 \pm 0.7 \times 10^7$ s^{-1} and T_1: $k_{T1} = 2.9 \pm 0.5 \times 10^4$ s^{-1}.

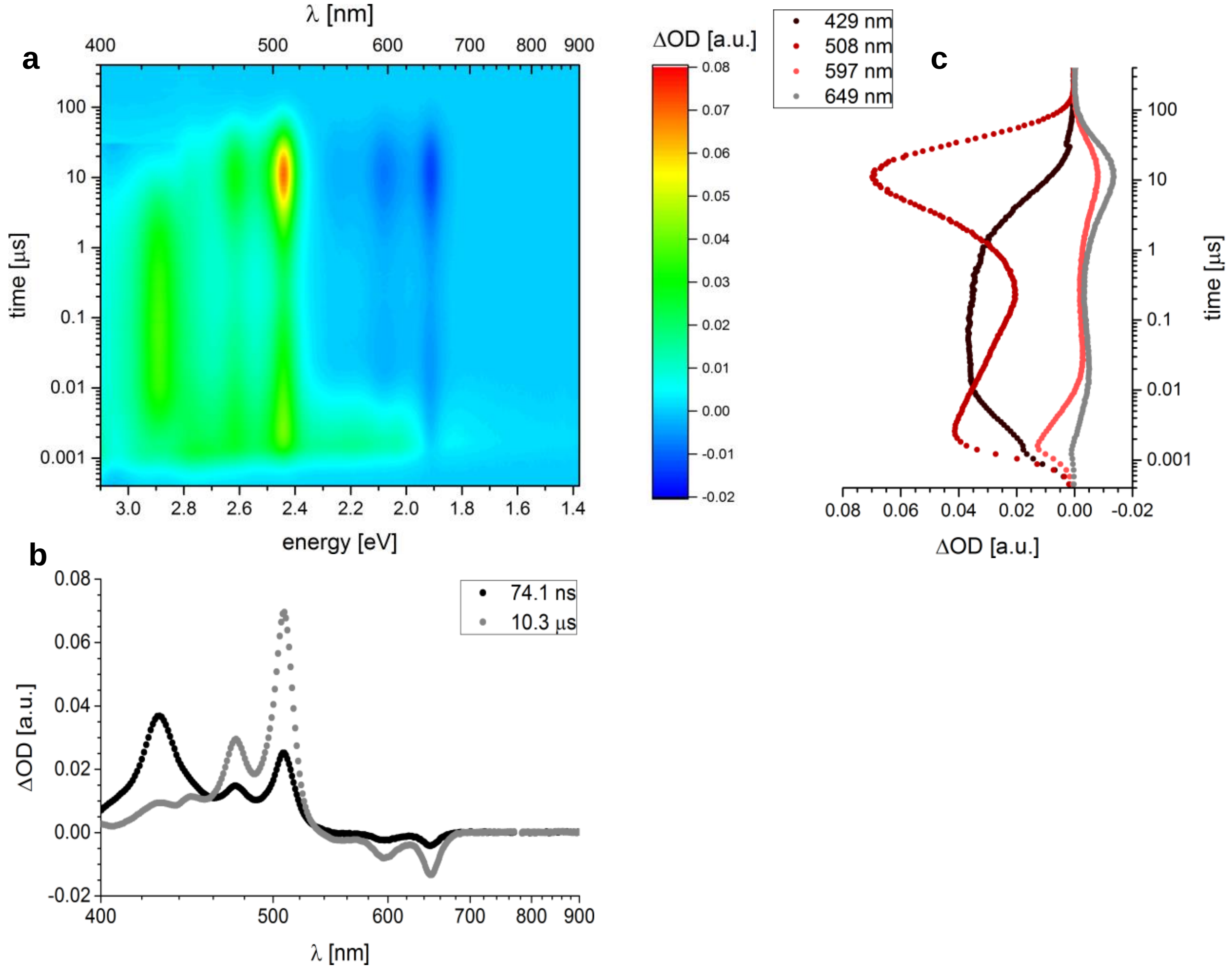

Supplementary Figure 15 | Transient absorption measurements. a) Differential absorption changes obtained upon nanosecond pump probe experiments (387 nm) of anthracene (1.0×10^{-3} M) and **NC** (5.0×10^{-5} M) in argon-saturated benzonitrile at room temperature with several time delays between 0 and 400 μs. b) Differential absorption spectra (visible and near-infrared) of the spectra shown in a) with time delays of 74.1 ns and 10.3 μs representing the triplet excited state of **NC**. c) Transient differential absorption kinetics at 429 nm (black), 508 nm (red), 597 nm (orange), and 649 nm (grey) illustrating the dynamics of the anthracene singlet excited state formation followed by the intersystem crossing to the corresponding anthracene triplet excited state and triplet-triplet energy transfer to **NC**.

Supplementary Methods 3 | Analysis of NC Transient Absorption Data at 295 K.

The nsTA data for **NC** in liquid benzonitrile at 295 K were first fit using a sum of four exponentials convoluted with a Gaussian instrument response to obtain the effective rate constants for each state in the unified kinetic model (Supplementary Fig. 16). The nsTA data were then globally fit using the unified kinetic model established by the TA and TREPR measurements of **NC** in solid butyronitrile at 105 K which required the same number of effective rate constants. The total deactivation of each state in the unified kinetic model was fixed to the corresponding effective rate constants obtained above, while the individual rate constants were allowed to vary. The fit was deemed satisfactory when (i) the species-associated spectra of $^1(T_1T_1)$, $^5(T_1T_1)$, and $2 \times T_1$ were of similar intensity, (ii) the yield of uncorrelated triplets predicted by the model matched that observed by nsTA spectroscopy, and (iii) the kinetic traces at 472 nm, 507 nm, and 650 nm were well fit by the kinetic model.

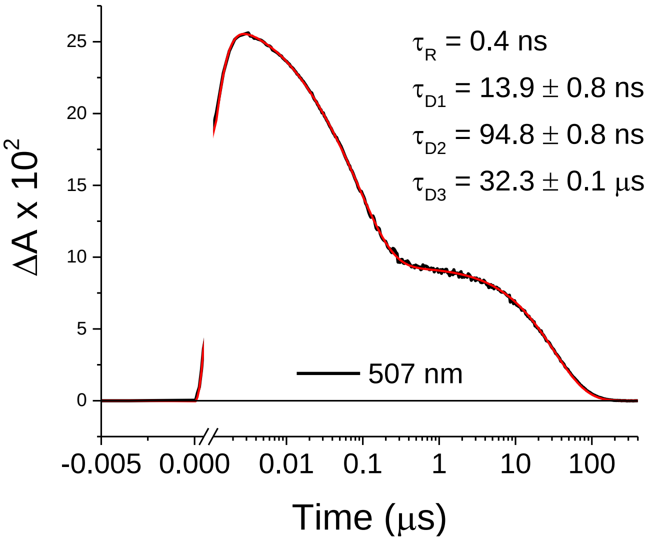

Supplementary Figure 16 | Transient absorption measurements. Transient absorption kinetics at 507 nm for **NC** in liquid benzonitrile at 295 K fit using a sum of four exponentials convoluted with a Gaussian instrument response to obtain the effective rate constants for each state in the unified kinetic model (Fig. 3).

Supplementary Methods 4 | Computational Details.

We have investigated different conformers of **NC** using density functional theory. Specifically, we have found the global minimum for the **NC** system using different methods. This resulted in three different conformers **NC1**, **NC2**, and **NC3** shown in Supplementary Fig. 17. At the M06-2X/6-31G(d) level of theory,[6, 7] the conformer **NC2** is more stable than conformers **NC1** (which is found to be a saddle point with a small imaginary frequency of –6.2 cm^{-1}) and **NC3** by 2.1 and 3.2 kcal mol^{-1}, respectively. At the B3LYP-D3/6-31G(d) level of theory, **NC2** is more stable than **NC1** by 4.1 kcal mol^{-1} (**NC3** could not be found at this level of theory). Therefore, we have used the **NC1** and **NC2** geometries obtained at the B3LYP-D3/6-31G(d) level of theory and the **NC3** at the M06-2X/6-31G(d) level of theory to have a full characterization of the effect of the relative conformation of the pentacene moieties in SF. Classical molecular-dynamics simulations in explicit benzonitrile solvent indicate that NC is conformationally labile with no clearly defined equilibrium structure.

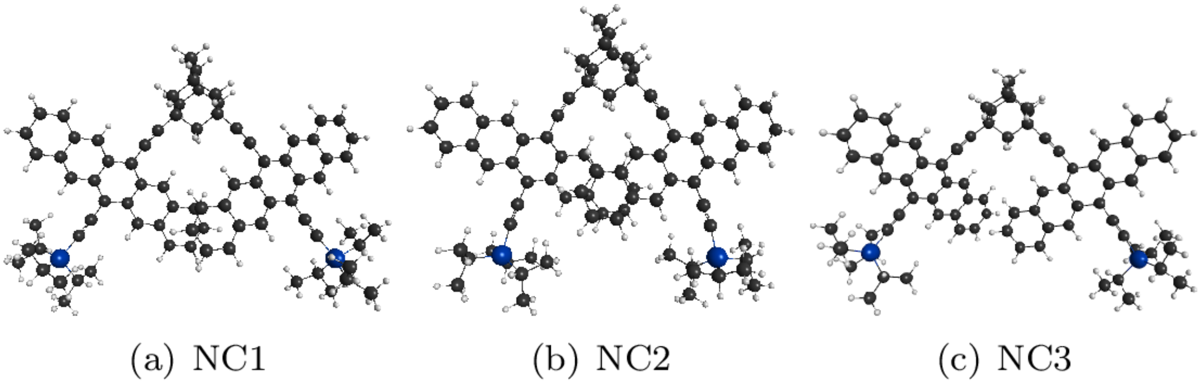

Supplementary Figure 17 | Molecular structures. Conformers **NC1**, **NC2**, and **NC3** of **NC**.

We have calculated the first fifteen low lying singlet, triplet and quintet excited states by employing state averaged complete active space self-consistent field (SA-CASSCF) and extended multi configuration quasi degenerate perturbation theory (XMCQDPT) methods. The active space 8 electrons in 8 orbitals (8e/8o) employed in the calculations, includes two highly occupied (HOMO–1 and HOMO) and two lowest unoccupied (LUMO and LUMO+1) molecular orbitals of each pentacene unit. As the charge-transfer states appear high in energy at the SA-CASSCF level of theory, a large number of states (15) are included in the average to calculate the wavefunction. The calculated vertical excitation energies, dipole moments, and oscillator strengths of the lowest lying singlet excited electronic states for each conformer are shown in Supplementary Table 1. The characterization of the electronic states as multi-excitonic, local excitation and charge transfer states is shown in Supplementary Figs 18-20 for **NC1**, **NC2**, and **NC3**, respectively.

To gain insight into the SF mechanism, we have investigated the diabatic electronic states relevant for the process and their interstate couplings.[8] These diabatic electronic states can be obtained by a suitable unitary transformation of the adiabatic electronic states. Among the different methods available, in this work we have employed the four-fold-way diabatization method of Truhlar and co-workers.[9, 10] In the following, first we will briefly describe the procedure of the four-fold-way and then we will present our results. The four-fold-way is based on the configurational uniformity concept proposed by Ruedenberg and co-workers.[11, 12] In this work, the adiabatic wavefunctions used in the diabatization process have been built using all configuration state functions (CSFs) whose coefficients are more than 0.20 in any of the adiabatic electronic states considered. The adiabatic (canonical) molecular orbitals of the active space that define the CSFs are then rotated such that they satisfy the so called three-fold density

criterion and MORMO conditions to form diabatic molecular orbitals (DMOs).[9, 10] The adiabatic states are then re-expressed by CSFs defined by DMOs and are transformed to diabatic states by applying a unitary transformation.

To reduce the computational cost in calculating the diabatic electronic states, we have employed a 4 electron in 4 orbital (4e/4o) active space, which has proved to give accurate results.[13, 14] The eight low-lying singlet excited electronic states of **NC1**, **NC2**, and **NC3** calculated at SA8-XMCQDPT(4, 4)/DZV level of theory, at their respective equilibrium structure (see Supplementary Table 2) were included in the diabatization process. These states have been used to build the diabatic states usually considered to play a role in SF, namely the singlet ground state $|^1(S_0S_0)>$, the correlated triplet pair (or ME) state $|^1(T_1T_1)>$, the locally excited (in one pentacene chromophore) singlet states $|^1(S_1S_0)>$ and $|^1(S_0S_1)>$ and the charge transfer states $|^1(CA)>$ and $|^1(AC)>$, where one TIPS-pentacene is formally a radical cation ($C^{\bullet+}$) and the other is a radical anion ($A^{\bullet-}$).[8, 13-15] In addition, and due to the energies and nature of the eight lowest-lying singlet excited electronic (adiabatic) states, two doubly excited states, $|^1(DE_1)>$ and $|^1(DE_2)>$ corresponding to double excitations between TIPS-pentacene units have also been included in the diabatic basis). We have selected 10 CSFs whose coefficients are more than 0.20 for all these three conformers (**NC1**, **NC2**, and **NC3**). Maximally localized DMOs were obtained in the calculations. The weights of the dominant CSFs obtained from the DMOs in the adiabatic states S_0, S_1, S_2, S_3, S_4, S_5, S_6 and S_7 for **NC1** were 100.0%, 100.0%, 95.5%, 94.3%, 100.0%, 100.0%, 99.4% and 99.4% respectively. For **NC2**, these weights were 99.9%, 98.6%, 95.9%, 94.2%, 98.7%, 98.7%, 99.4% and 99.5%. Similarly, for **NC3**, these weights were 100.0%, 100.0%, 94.8%, 93.7%, 99.9%, 99.9%, 99.3% and 99.4%. It can be seen that the contributions from the dominant CSFs are ~94% or more for **NC1**, **NC2**, and **NC3**. The electronic

Hamiltonian in the diabatic basis defined by the relevant states of **NC1, NC2,** and **NC3** are collected in Supplementary Tables 3, 4, and 5, respectively.

The adiabatic electronic states (S_0, S_1, S_2, S_3, S_4, S_5, S_6 and S_7) are the linear combination of the diabatic states ($|^1(S_0S_0)>$, $|^1(T_1T_1)>$, $|^1(S_1S_0)>$, $|^1(S_0S_1)>$, $|^1(DE_1)>$, $|^1(DE_2)>$ $|^1(CA)>$ and $|^1(AC)>$). The adiabatic electronic states of **NC1**, **NC2**, and **NC3** in terms of the respective diabatic electronic states are presented in equations (1), (2) and (3), respectively.

$$\begin{pmatrix} |S_0> \\ |S_1> \\ |S_2> \\ |S_3> \\ |S_4> \\ |S_5> \\ |S_6> \\ |S_7> \end{pmatrix} = \begin{pmatrix} 0.94 & 0.00 & -0.02 & -0.02 & 0.24 & -0.23 & 0.01 & -0.01 \\ 0.00 & 1.00 & 0.00 & 0.00 & 0.00 & 0.00 & 0.05 & -0.06 \\ 0.00 & 0.00 & 0.72 & -0.69 & -0.02 & -0.02 & 0.00 & 0.00 \\ 0.07 & 0.00 & 0.68 & 0.72 & -0.07 & 0.07 & 0.01 & -0.01 \\ -0.33 & 0.01 & 0.08 & 0.09 & 0.68 & -0.64 & -0.04 & 0.03 \\ 0.00 & 0.00 & 0.01 & -0.02 & 0.68 & 0.74 & -0.02 & -0.02 \\ -0.02 & -0.05 & 0.00 & -0.01 & 0.03 & 0.00 & 0.99 & 0.08 \\ 0.02 & 0.06 & 0.01 & 0.00 & -0.01 & 0.03 & -0.08 & 0.99 \end{pmatrix} \begin{pmatrix} |\,^1(S_0S_0)> \\ |\,^1(T_1T_1)> \\ |\,^1(S_1S_0)> \\ |\,^1(S_0S_1)> \\ |\,^1(DE_1)> \\ |\,^1(DE_2)> \\ |\,^1(CA)> \\ |\,^1(AC)> \end{pmatrix} \qquad (1)$$

$$\begin{pmatrix} |S_0> \\ |S_1> \\ |S_2> \\ |S_3> \\ |S_4> \\ |S_5> \\ |S_6> \\ |S_7> \end{pmatrix} = \begin{pmatrix} 0.94 & 0.00 & -0.02 & -0.01 & -0.24 & 0.23 & -0.02 & -0.02 \\ -0.01 & -0.95 & -0.09 & -0.12 & -0.03 & 0.03 & 0.19 & 0.18 \\ 0.01 & -0.02 & 0.75 & -0.63 & 0.03 & 0.01 & 0.06 & -0.18 \\ 0.07 & -0.19 & 0.62 & 0.74 & 0.10 & -0.10 & -0.09 & -0.01 \\ 0.32 & -0.05 & -0.14 & -0.12 & 0.67 & -0.63 & -0.09 & -0.09 \\ 0.00 & 0.00 & 0.01 & 0.00 & -0.68 & -0.72 & 0.07 & -0.08 \\ 0.01 & 0.03 & -0.08 & 0.16 & 0.08 & 0.06 & 0.77 & -0.60 \\ -0.08 & -0.24 & -0.13 & 0.00 & -0.09 & 0.10 & -0.58 & -0.75 \end{pmatrix} \begin{pmatrix} |\,^1(S_0S_0)> \\ |\,^1(T_1T_1)> \\ |\,^1(S_1S_0)> \\ |\,^1(S_0S_1)> \\ |\,^1(DE_1)> \\ |\,^1(DE_2)> \\ |\,^1(CA)> \\ |\,^1(AC)> \end{pmatrix} \qquad (2)$$

$$\begin{pmatrix} |S_0> \\ |S_1> \\ |S_2> \\ |S_3> \\ |S_4> \\ |S_5> \\ |S_6> \\ |S_7> \end{pmatrix} = \begin{pmatrix} 0.93 & 0.00 & 0.02 & 0.02 & -0.25 & 0.25 & 0.00 & 0.00 \\ 0.01 & 1.00 & 0.00 & 0.00 & 0.00 & 0.00 & 0.00 & 0.00 \\ -0.02 & 0.00 & 0.79 & -0.60 & -0.04 & 0.01 & 0.03 & 0.02 \\ -0.08 & 0.00 & 0.60 & 0.79 & -0.08 & 0.07 & -0.01 & 0.00 \\ 0.35 & 0.00 & 0.12 & 0.07 & 0.69 & -0.62 & 0.00 & 0.00 \\ -0.01 & 0.00 & 0.02 & 0.00 & 0.67 & 0.74 & 0.00 & 0.00 \\ 0.00 & 0.03 & -0.02 & 0.03 & 0.00 & 0.00 & 1.00 & 0.00 \\ 0.00 & -0.01 & -0.02 & 0.01 & 0.00 & 0.00 & 0.00 & 1.00 \end{pmatrix} \begin{pmatrix} |\,^1(S_0S_0)> \\ |\,^1(T_1T_1)> \\ |\,^1(S_1S_0)> \\ |\,^1(S_0S_1)> \\ |\,^1(DE_1)> \\ |\,^1(DE_2)> \\ |\,^1(CA)> \\ |\,^1(AC)> \end{pmatrix} \qquad (3)$$

To quantify the contribution of the direct and mediated mechanism to the overall SF process we have calculated the effective coupling, V_{eff} , of $|^1(S_1S_0)>$ to $|^1(T_1T_1)>$ (or $|^1(S_0S_1)>$ to $|^1(T_1T_1)>$):[16, 17]

$$V_{eff} = V_{^1(S_1S_0),\ ^1(T_1T_1)} - 2\frac{V_{^1(S_1S_0),\ ^1(CA)}V_{^1(CA),\ ^1(T_1T_1)} + V_{^1(S_1S_0),\ ^1(AC)}V_{^1(AC),\ ^1(T_1T_1)}}{[E(CT) - E(\ ^1(T_1T_1))] + [E(CT) - E(\ ^1(S_1S_0))]} \quad (4)$$

In this expression, $E(i)$ are the energies of the corresponding diabatic states [we have used for E(CT) the mean of E(^{1}(CA)) and E(^{1}(AC))], $V_{i,j}$ are the couplings between the states i and j and it is assumed that the mixing of the CT states with $|^1(T_1T_1)>$ and $|^1(S_1S_0)>$ $|^1(S_0S_1)>$) can be described perturbatively. The contributions of the direct and mediated (*via* CT states) mechanisms are accounted for by the first and second term on the right hand side, respectively.

To substantiate the hypothesis of the involvement of a quintet state in the singlet fission mechanism, we have calculated the energy of the lowest-lying quintet state of $|^5(T_1T_1)>$ type and shown that this state is nearly degenerate with $|^1(T_1T_1)>$. This indicates that a mixed singlet-quintet spin state is formed in the singlet fission process. Specifically, for a system of two coupled triplet states, without external magnetic field and neglecting for simplicity spin-orbit coupling, the spin part of the full Hamiltonian (H_s) originates from the spin-spin exchange and magnetic dipole-dipole interaction between the electrons of the two triplet centers. This can be expressed as [18, 19]

$$H_s = H_{zf} + H_{eei} \quad (5)$$

where H_{zf}, and H_{eei} are the zero-field and electron-electron interactions, respectively that have the general form

$$H_{zf} = hS^T DS \quad (6)$$

$$H_{eei} = h{S_1}^T JS_2 \quad (7)$$

where h is Planck´s constant, S_i is the spin at chromophore i, D is the zero-field splitting tensor and J is the exchange coupling tensor. Diagonalization of the full Hamiltonian (i.e. the sum of the electrostatic and magnetic terms) generates nine eigenfunctions that are linear combinations of singlet ($|S\rangle$), triplet ($|T\rangle$), and quintet ($|Q\rangle$) spin states.[8, 20] When no external magnetic field is present, the spin basis set can be built up from all pair combinations of zero field triplet spin functions of each chromophoric site.[8, 18-20]

Employing this basis, and assuming the energies of $|^1(T_1T_1)>$ and $|^5(T_1T_1)>$ to be nearly degenerate, it can be shown[19] that the triplet pair state of singlet character $|^1(T_1T_1)>$ formed by singlet fission is not an energy eigenstate of the full Hamiltonian. Instead, two (or three, depending on the inter-triplet exchange interaction strength strong or weak, respectively) states with mixed singlet-quintet character are formed that are a mixture of the states $|^1(T_1T_1)>$ and $|^5(T_1T_1)>$. Therefore, population of $|^5(T_1T_1)>$ through spin mixing is possible. The emergence of a quintet channel in singlet fission is then an intrinsic characteristic of the particular coupling regime. It requires that the lowest-lying triplet pair states of singlet and quintet character are quasi degenerate, i.e. have energy differences that are similar or smaller than the spin coupling.

Supplementary Table 1 | Computed molecular properties. Vertical excitation energy (ΔE in eV),[a] modulus of the dipole moment (μ, D),[b] and oscillator strength (f),[b] for the lowest-lying singlet, triplet and quintet excited states of **NC1**, **NC2**, and **NC3**.

NC1				NC2				NC3			
State	ΔE	μ	f	State	ΔE	μ	f	State	ΔE	μ	f
S_0	0.00	0.90	-	S_0	0.00	0.36	-	S_0	0.00	1.22	-
T_1	0.96	0.66	0.000	T_1	0.94	0.17	0.000	T_1	0.91	1.29	0.000
T_2	1.01	0.75	0.000	T_2	0.96	0.39	0.000	T_2	0.92	1.40	0.000
S_1	2.00	0.55	<0.001	S_1	1.90	0.18	<0.001	S_1	1.87	1.50	<0.001
S_2	2.00	4.99	0.423	T_3	1.92	0.18	0.000	T_3	1.87	1.50	0.000
$Q_1 \equiv {}^5(T_1T_1)$	2.00	0.55	0.000	S_2	1.94	3.49	0.250	Q_1	1.88	1.49	0.000
T_3	2.01	0.55	0.000	Q_1	1.95	0.18	0.000	S_2	1.95	4.92	0.389
S_3	2.04	5.42	0.469	S_3	1.96	3.22	0.668	S_3	1.98	4.25	0.506
S_4	2.26	3.67	0.040	S_4	2.19	3.74	0.047	S_4	2.16	1.65	0.071
S_5	2.46	4.40	0.056	T_4	2.30	5.26	0.000	S_5	2.35	5.18	0.017
T_4	2.57	0.95	0.000	T_5	2.33	5.66	0.000	T_4	2.51	0.93	0.000
T_5	2.63	0.58	0.000	S_5	2.33	6.89	0.055	T_5	2.59	1.29	0.000
T_6	2.72	37.36	0.000	S_6	2.35	7.62	0.005	T_6	2.70	46.78	0.000
S_6	2.74	41.29	0.003	S_7	2.40	2.10	0.015	S_6	2.72	46.69	<0.001
S_7	2.74	41.05	0.006	T_6	2.55	0.46	0.000	T_7	2.98	48.42	0.000
T_7	2.75	33.45	0.000	T_7	2.60	0.52	0.000	S_7	2.99	48.47	<0.001

[a] Calculated at SA15-XMCQDPT(8,8)/DZV level of theory

[b] Calculated at the CASSCF level of theory.

Supplementary Table 2 | Computed molecular properties. Vertical excitation energy (ΔE in eV),[a] modulus of the dipole moment (μ, D),[b] oscillator strength (f),[b] and character for the lowest lying singlet excited states (char.)[c] of **NC1**, **NC2**, and **NC3**.

State	NC1				NC2				NC3			
	ΔE	μ	f	char.	ΔE	μ	f	Char.	ΔE	μ	f	char.
S_1	1.72	0.80	<0.001	ME	1.67	0.17	<0.001	ME	1.62	1.23	<0.001	ME
S_2	1.83	0.38	0.380	LE	1.79	0.91	0.160	LE	1.78	1.65	0.442	LE
S_3	1.84	0.38	0.730	LE	1.79	0.98	0.900	LE	1.81	1.50	0.640	LE
S_4	2.00	1.16	0.001	DE	1.94	0.47	0.003	DE	1.91	0.77	<0.001	DE
S_5	2.21	1.04	<0.001	DE	2.15	0.40	<0.001	DE	2.13	1.44	0.001	DE
S_6	2.51	44.20	0.007	CT	2.24	26.05	0.006	CT	2.51	46.90	<0.001	CT
S_7	2.55	44.23	0.006	CT	2.28	26.05	0.002	CT	2.78	48.76	<0.001	CT

[a] Calculated at the SA8-XMCQDPT(4,4)/DZV level of theory.

[b] Calculated at the CASSCF level of theory.

[c] Character of excited state: ME = multiexcitonic state, LE = excited states that correlate with the plus and minus combinations of locally excited states of both pentacene monomers, CT = charge transfer states, DE = doubly excited states.

Supplementary Table 3 | Computed molecular properties. Energies and coupling matrix elements (meV) of the low-lying diabatic electronic states of **NC1** calculated at the XMCQDPT/DZV level of theory.[a]

**NC1**	**$|^1(S_0S_0)>$**	**$|^1(T_1T_1)>$**	**$|^1(S_1S_0)>$**	**$|^1(S_0S_1)>$**	**$|^1(DE_1)>$**	**$|^1(DE_2)>$**	**$|^1(CA)>$**	**$|^1(AC)>$**
**$|^1(S_0S_0)>$**	0	0	–31	32	–462	452	–35	30
**$|^1(T_1T_1)>$**	0	1514	0	0	0	0	–42	49
**$|^1(S_1S_0)>$**	–31	0	1640	–8	–1	10	–2	–8
**$|^1(S_0S_1)>$**	32	0	–8	1642	10	–1	–5	1
**$|^1(DE_1)>$**	–462	0	–1	10	1781	212	10	–2
**$|^1(DE_2)>$**	452	0	10	–1	212	1799	–2	9
**$|^1(CA)>$**	–35	–42	–2	–5	10	–2	2309	0
**$|^1(AC)>$**	30	49	–8	1	–2	9	0	2352

[a] Calculated at the XMCQDPT/DZV level of theory (8 roots with equal weights and a 4e-/4o active space were used in the CASSCF calculation).

Supplementary Table 4 | Computed molecular properties. Energies and coupling matrix elements (meV) of the low-lying diabatic electronic states of **NC2** calculated at the XMCQDPT/DZV level of theory.[a]

**NC2**	**$\|^1(S_0S_0)>$**	**$\|^1(T_1T_1)>$**	**$\|^1(S_1S_0)>$**	**$\|^1(S_0S_1)>$**	**$\|^1(DE_1)>$**	**$\|^1(DE_2)>$**	**$\|^1(CA)>$**	**$\|^1(AC)>$**
**$\|^1(S_0S_0)>$**	0	–2	25	18	436	–426	62	53
**$\|^1(T_1T_1)>$**	–2	1496	5	–11	7	–3	101	99
**$\|^1(S_1S_0)>$**	25	5	1584	–2	–22	8	13	74
**$\|^1(S_0S_1)>$**	18	–11	–2	1584	–11	23	58	–41
**$\|^1(DE_1)>$**	436	7	–22	–11	1717	213	19	13
**$\|^1(DE_2)>$**	–426	–3	8	23	213	1735	–10	19
**$\|^1(CA)>$**	62	101	13	58	19	–10	2008	–4
**$\|^1(AC)>$**	53	99	74	–41	13	19	–4	2009

[a] Calculated at the XMCQDPT/DZV level of theory (8 roots with equal weights and a 4e-/4o active space were used in the CASSCF calculation).

Supplementary Table 5 | Computed molecular properties. Energies and coupling matrix elements (meV) of the low-lying diabatic electronic states of **NC3** calculated at the XMCQDPT/DZV level of theory.[a]

**NC3**	**$\|^1(S_0S_0)>$**	**$\|^1(T_1T_1)>$**	**$\|^1(S_1S_0)>$**	**$\|^1(S_0S_1)>$**	**$\|^1(DE_1)>$**	**$\|^1(DE_2)>$**	**$\|^1(CA)>$**	**$\|^1(AC)>$**
**$\|^1(S_0S_0)>$**	0	0	–39	–32	452	–440	–1	–1
**$\|^1(T_1T_1)>$**	0	1380	0	0	–1	1	–1	–7
**$\|^1(S_1S_0)>$**	–39	0	1551	11	25	–15	–12	–18
**$\|^1(S_0S_1)>$**	32	0	11	1557	12	–15	20	14
**$\|^1(DE_1)>$**	452	–1	23	12	1645	232	–1	–2
**$\|^1(DE_2)>$**	–440	1	–15	–15	232	1674	–2	0
**$\|^1(CA)>$**	–1	–1	–12	20	–1	–2	2269	0
**$\|^1(AC)>$**	–1	–7	–18	14	–2	0	0	2540

[a] Calculated at the XMCQDPT/DZV level of theory (8 roots with equal weights and a 4e-/4o active space were used in the CASSCF calculation).

Supplementary Table 6 | Computed molecular properties. Direct, indirect, and total coupling (meV) of the lowest lying absorbing singlet states $|^1(S_1S_0)\rangle$ and $|^1(S_0S_1)\rangle$ to $|^1(T_1T_1)\rangle$ calculated at the XMQCDPT/DZV level of theory using Truhlar's four-fold-way diabatization method.

System	State	Direct	Mediated	Total
**NC1**	$|^1(S_1S_0)\rangle$	0.00	–0.17	–0.17
	$|^1(S_0S_1)\rangle$	0.00	–0.35	–0.35
**NC2**	$|^1(S_1S_0)\rangle$	5.00	–18.42	–13.42
	$|^1(S_0S_1)\rangle$	–11.00	–3.35	–14.35
**NC3**	$|^1(S_1S_0)\rangle$	–0.11	–0.17	–0.28
	$|^1(S_0S_1)\rangle$	–0.08	–0.15	0.23

State	Orbital transition	Weight	Character
S_1	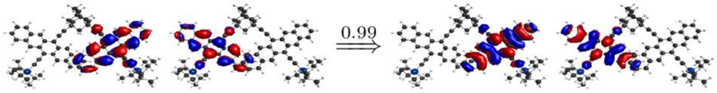	0.99	ME
S_2	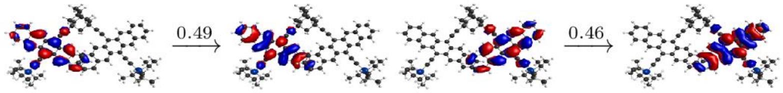	0.95	LE
S_3	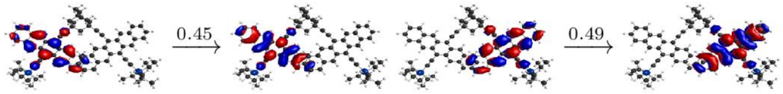	0.94	LE
S_4	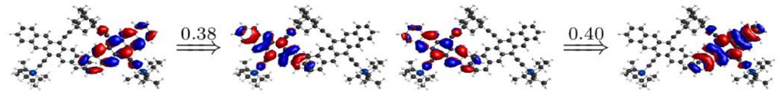	0.78	DE
S_5	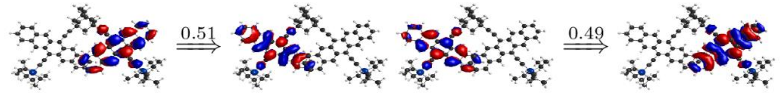	1.00	DE
S_6	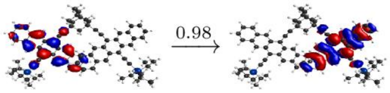	0.98	CT
S_7	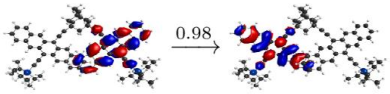	0.98	CT

Supplementary Figure 18 | Electronic excitations and related orbitals. Characterization of the adiabatic excited electronic states of **NC1** in terms of the electron excitations involving localized CASSCF molecular orbitals.[a, b, c]

[a] Double arrows represent double excitations and single arrows represent single excitations.

[b] The number on each arrow is the weight of the particular excitation in the wavefunction of the adiabatic state.

[c] Character of excited state: ME = multiexcitonic state, LE = excited states that correlate with the plus and minus combinations of locally excited states of both pentacene monomers, CT = charge transfer states, DE = doubly excited states.

State	Orbital transition	Weight	Character
S_1	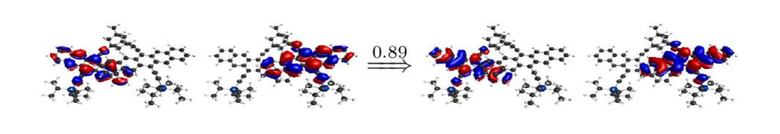	0.89	ME
S_2	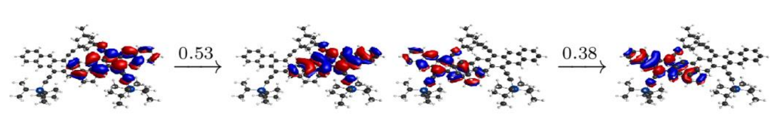	0.91	LE
S_3	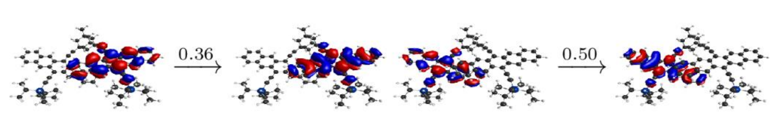	0.86	LE
S_4	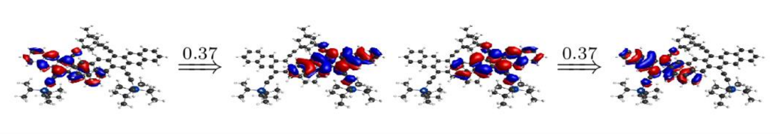	0.74	DE
S_5	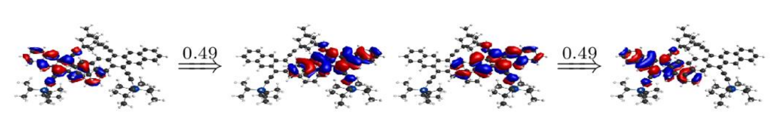	0.94	DE
S_6	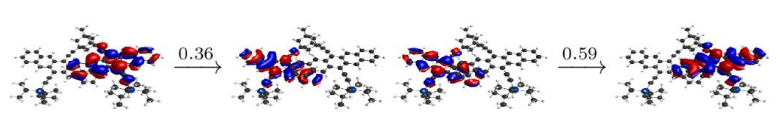	0.95	CT
S_7	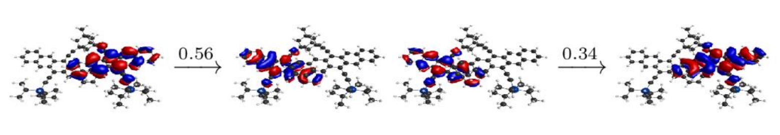	0.90	CT

Supplementary Figure 19 | Electronic excitations and related orbitals. Characterization of the adiabatic excited electronic states of **NC2** in terms of electron excitations involving localized CASSCF molecular orbitals.[a, b, c]

[a] Double arrows represent double excitations and single arrows represent single excitations.

[b] The number on each arrow is the weight of the particular excitation in the wavefunction of the adiabatic state.

[c] Character of excited state: ME = multiexcitonic state, LE = excited states that correlate with the plus and minus combinations of locally excited states of both pentacene monomers, CT = charge transfer states, DE = doubly excited states.

State	Orbital transition	Weight	Character
S_1	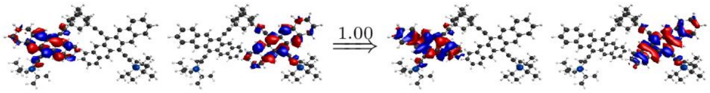	1.00	ME
S_2	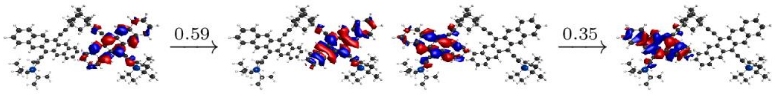	0.94	LE
S_3	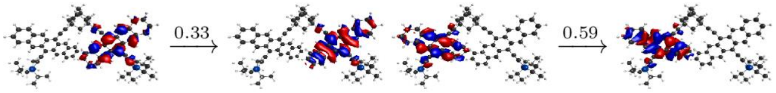	0.92	LE
S_4	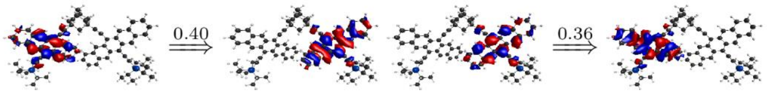	0.76	DE
S_5	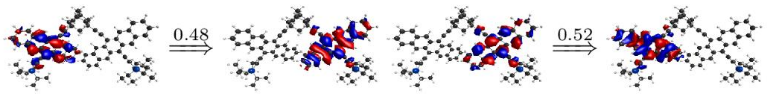	1.00	DE
S_6	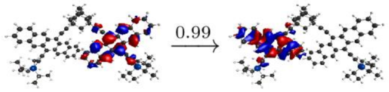	0.99	CT
S_7	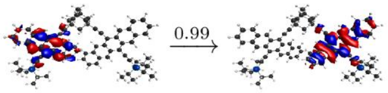	0.99	CT

Supplementary Figure 20 | Electronic excitations and related orbitals. Characterization of the adiabatic excited electronic states of **NC3** in terms of electron excitations involving localized CASSCF molecular orbitals. [a, b, c]

[a] Double arrows represent double excitations and single arrows represent single excitations.

[b] The number on each arrow is the weight of the particular excitation in the wavefunction of the adiabatic state.

[c] Character of excited state: ME = multiexcitonic state, LE = excited states that correlate with the plus and minus combinations of locally excited states of both pentacene monomers, CT = charge transfer states, DE = doubly excited states.

Supplementary Table 7 | Cartesian coordinates (in Ångstroms).

NC1 Cartesian coordinates:

C	2.409682	4.029745	0.222222
C	3.227822	3.133650	0.291510
C	4.083302	2.002163	0.352076
C	3.532465	0.749466	0.750340
C	4.364127	-0.439032	0.731634
C	5.728921	-0.335298	0.343952
C	6.286712	0.921338	-0.026212
C	5.449514	2.105738	-0.026450
C	3.787077	-1.677636	1.085999
C	2.451942	-1.791744	1.456729
C	1.627718	-0.600087	1.504331
C	2.184506	0.623078	1.151325
C	0.250869	-0.730659	1.883296
C	-0.281053	-1.953100	2.180471
C	0.531773	-3.130425	2.129186
C	1.851856	-3.052255	1.784233
C	6.016000	3.338629	-0.414980
C	7.349420	3.451821	-0.791640
C	8.187076	2.267725	-0.788631
C	7.638118	1.048099	-0.410345
C	9.560641	2.396016	-1.180061
C	10.072947	3.607810	-1.549948
C	9.246269	4.777682	-1.553228
C	7.931822	4.701392	-1.186799
C	6.545643	-1.492436	0.308578
H	4.411162	-2.566422	1.061447
H	1.564090	1.512984	1.178126
H	-0.369953	0.161399	1.896523

H	-1.332073	-2.042294	2.438905
H	0.084531	-4.092881	2.361575
H	2.470498	-3.945603	1.743235
H	5.383230	4.221589	-0.415919
H	8.264583	0.161583	-0.411665
H	10.183824	1.504752	-1.175453
H	11.115134	3.693502	-1.845266
H	9.676018	5.730238	-1.851039
H	7.301915	5.587769	-1.188365
C	7.273185	-2.474452	0.261552
Si	8.445770	-3.880122	0.115124
C	8.569289	-4.798808	1.790800
C	8.323780	-3.894127	3.013048
C	9.902161	-5.561200	1.936035
C	7.765340	-5.034666	-1.245679
C	6.335793	-5.517837	-0.940006
C	8.698092	-6.223866	-1.540934
C	10.121324	-3.135392	-0.421032
C	10.041754	-2.513895	-1.828432
C	10.651457	-2.102489	0.591862
H	7.761035	-5.545925	1.766998
H	7.357813	-3.382151	2.953670
H	9.095976	-3.120476	3.102809
H	8.342284	-4.482023	3.940999
H	10.090907	-6.243786	1.099722
H	9.908224	-6.159189	2.857308
H	10.752086	-4.870445	1.994230
H	7.716715	-4.408616	-2.148925
H	5.936303	-6.112073	-1.773140
H	5.652322	-4.678018	-0.769440
H	6.309848	-6.157128	-0.047856

H	9.719216	-5.902870	-1.780534
H	8.328093	-6.810020	-2.393030
H	8.756102	-6.905853	-0.683488
H	10.835027	-3.972923	-0.457185
H	11.008203	-2.079582	-2.119042
H	9.296016	-1.709315	-1.864223
H	9.769691	-3.250230	-2.593315
H	10.803946	-2.535544	1.586509
H	9.953280	-1.263659	0.704254
H	11.614230	-1.689217	0.261032
C	-0.181540	6.718734	1.192203
C	1.079888	5.834424	1.332631
C	1.267682	4.933093	0.082962
C	1.395732	5.833783	-1.174416
C	0.130644	6.701462	-1.323208
C	-0.034922	7.589714	-0.075909
C	-1.417225	5.804989	1.021279
C	-1.272062	4.901514	-0.232340
C	-1.110216	5.801207	-1.486803
C	0.008504	4.039607	-0.074583
C	-0.347110	7.597463	2.436218
H	0.998989	5.203811	2.227422
H	1.967642	6.466580	1.464245
H	1.537273	5.203332	-2.060882
H	2.286273	6.467986	-1.080953
H	0.233166	7.333686	-2.214255
H	-0.919204	8.232731	-0.185340
H	0.833731	8.254023	0.031491
H	-2.324560	6.416059	0.928208
H	-1.544640	5.174520	1.910750
C	-2.394834	3.972146	-0.354385

H	-2.012022	6.412164	-1.617996
H	-1.013349	5.169553	-2.378389
H	-0.095572	3.390365	0.802400
H	0.128009	3.390857	-0.949848
C	-3.200532	3.063392	-0.398881
C	-4.048642	1.925007	-0.422452
H	-0.450932	6.985576	3.340759
H	-1.239054	8.230661	2.353316
H	0.521181	8.254074	2.571502
C	-5.445920	2.058992	-0.197504
C	-6.285816	0.876902	-0.168167
C	-5.699305	-0.407373	-0.351537
C	-4.302273	-0.542513	-0.585193
C	-3.466775	0.642285	-0.638808
C	-7.670873	1.035130	0.049710
C	-8.249780	2.285479	0.234686
C	-7.409434	3.467430	0.210126
C	-6.043035	3.322414	-0.001059
C	-8.023379	4.748839	0.403980
C	-9.370587	4.857507	0.606914
C	-10.200353	3.690095	0.629939
C	-9.658093	2.448527	0.450269
C	-2.088560	0.484719	-0.903182
C	-1.509437	-0.763746	-1.096106
C	-2.338606	-1.949497	-1.004237
C	-3.700226	-1.806911	-0.761729
C	-1.718093	-3.232338	-1.164292
C	-0.376460	-3.335814	-1.403449
C	0.439975	-2.164331	-1.507043
C	-0.109382	-0.922359	-1.362037
C	-6.524687	-1.557994	-0.308625

H	-8.299761	0.150467	0.072463
H	-5.408418	4.203796	-0.021478
H	-7.390843	5.633146	0.385038
H	-9.824359	5.834078	0.752430
H	-11.269058	3.801638	0.791735
H	-10.284057	1.559404	0.465266
H	-1.463839	1.370322	-0.959449
H	-4.326738	-2.692334	-0.703430
H	-2.340404	-4.120746	-1.087514
H	0.084941	-4.313437	-1.512165
H	1.506634	-2.271548	-1.679850
H	0.513391	-0.033236	-1.412195
C	-7.262324	-2.533133	-0.275813
Si	-8.437641	-3.943551	-0.248774
C	-9.990124	-3.464084	-1.267413
C	-10.296114	-1.954079	-1.253870
C	-11.233238	-4.275329	-0.849492
C	-7.530959	-5.433561	-1.024672
C	-7.026176	-5.133882	-2.447807
C	-8.369535	-6.724871	-0.996950
C	-8.873401	-4.306677	1.573436
C	-7.662102	-4.860749	2.346858
C	-9.449770	-3.069459	2.287950
H	-9.745794	-3.739869	-2.304726
H	-9.448900	-1.364590	-1.618728
H	-10.530367	-1.601107	-0.241497
H	-11.165301	-1.727069	-1.886282
H	-11.547393	-4.026135	0.171323
H	-11.061302	-5.357171	-0.884769
H	-12.081027	-4.054877	-1.512095
H	-6.649047	-5.588927	-0.384943

H	-6.399885	-4.235006	-2.476289
H	-7.860707	-4.979018	-3.143980
H	-6.432777	-5.971851	-2.838362
H	-7.784260	-7.580499	-1.360160
H	-9.249915	-6.640503	-1.646266
H	-8.722471	-6.971094	0.011973
H	-9.652781	-5.084108	1.554590
H	-7.923873	-5.055164	3.395892
H	-6.831606	-4.143370	2.344385
H	-7.292454	-5.800763	1.920860
H	-9.690631	-3.299034	3.334885
H	-10.367602	-2.706423	1.811958
H	-8.727635	-2.243708	2.288582

NC2 Cartesian coordinates:

C	2.372728	4.646447	0.313315
C	3.046683	3.638736	0.414874
C	3.675672	2.366943	0.498251
C	2.874735	1.237283	0.839437
C	3.454708	-0.091095	0.806356
C	4.828131	-0.248127	0.464516
C	5.638826	0.882423	0.167016
C	5.051367	2.208743	0.179240
C	2.627844	-1.199691	1.093569
C	1.283533	-1.057777	1.416175
C	0.708275	0.269942	1.471794
C	1.512000	1.366183	1.184493
C	-0.684502	0.406017	1.782268
C	-1.458506	-0.696504	2.015808
C	-0.887972	-2.008558	1.970290
C	0.436045	-2.183351	1.678760

C	5.865295	3.315978	-0.139842
C	7.211549	3.174306	-0.456462
C	7.799805	1.848130	-0.465985
C	7.004768	0.749464	-0.158538
C	9.189416	1.717456	-0.795069
C	9.946276	2.815809	-1.093049
C	9.365734	4.125074	-1.082847
C	8.045201	4.296677	-0.775360
C	5.370690	-1.555435	0.409103
H	3.057629	-2.195024	1.048311
H	1.077229	2.357735	1.219828
H	-1.118171	1.402242	1.799909
H	-2.518619	-0.585017	2.223676
H	-1.523354	-2.868873	2.158100
H	0.870071	-3.179149	1.632368
H	5.418674	4.306408	-0.131322
H	7.442897	-0.244656	-0.165926
H	9.627294	0.722141	-0.801619
H	10.998346	2.703467	-1.340582
H	9.987298	4.983483	-1.322663
H	7.600908	5.289119	-0.766221
C	5.768902	-2.711238	0.367698
Si	6.137484	-4.508646	0.345664
C	4.953866	-5.353954	-0.906291
C	3.613756	-4.615648	-1.088377
C	4.714626	-6.839845	-0.570531
C	7.956917	-4.733525	-0.181610
C	8.251689	-4.081719	-1.544770
C	8.399342	-6.208556	-0.167711
C	5.859691	-5.160906	2.118030
C	6.921748	-4.624252	3.095494

C	4.442738	-4.842771	2.633650
H	5.483422	-5.311772	-1.870310
H	3.762551	-3.568928	-1.371385
H	3.019810	-4.618984	-0.165739
H	3.006026	-5.099418	-1.865978
H	4.115440	-7.323484	-1.353850
H	4.165770	-6.951377	0.372292
H	5.648415	-7.405455	-0.474699
H	8.543433	-4.196074	0.578501
H	9.320448	-4.149059	-1.789520
H	7.969227	-3.022649	-1.559206
H	7.704182	-4.581486	-2.354422
H	8.223633	-6.686881	0.803655
H	9.470960	-6.299277	-0.391178
H	7.862917	-6.791271	-0.927258
H	5.965599	-6.255307	2.062643
H	6.744485	-5.002585	4.111463
H	6.893510	-3.528398	3.144861
H	7.937310	-4.919479	2.807009
H	4.297840	-5.236649	3.648794
H	3.661600	-5.277093	1.999104
H	4.273236	-3.759780	2.674014
C	-0.214814	7.404835	1.121044
C	1.030576	6.510807	1.339989
C	1.284806	5.607627	0.105823
C	1.497911	6.501386	-1.143367
C	0.250265	7.380729	-1.370376
C	0.018014	8.272904	-0.136414
C	-1.450398	6.504646	0.875460
C	-1.238515	5.601475	-0.366920
C	-0.991283	6.495610	-1.609411

C	0.024956	4.736829	-0.128825
C	-0.447355	8.287230	2.351729
H	0.889520	5.882104	2.228671
H	1.912000	7.138215	1.526104
H	1.685052	5.865975	-2.017862
H	2.386606	7.129086	-1.001085
H	0.414214	8.010334	-2.253975
H	-0.851181	8.924739	-0.300462
H	0.885326	8.928189	0.023836
H	-2.342819	7.127592	0.730770
H	-1.636633	5.875054	1.755208
C	-2.324833	4.634747	-0.556532
H	-1.874105	7.119334	-1.797518
H	-0.847151	5.861001	-2.492508
H	-0.145086	4.090695	0.738286
H	0.197336	4.086602	-0.992439
C	-2.996796	3.623643	-0.635196
C	-3.624197	2.349551	-0.691050
H	-0.610695	7.677828	3.249167
H	-1.327073	8.928030	2.214507
H	0.416925	8.936539	2.538115
C	-4.998960	2.196360	-0.365499
C	-5.583831	0.869637	-0.321410
C	-4.771462	-0.266134	-0.595267
C	-3.400342	-0.114288	-0.946719
C	-2.822243	1.213870	-1.009028
C	-6.948045	0.741696	0.013382
C	-7.743522	1.845569	0.300483
C	-7.157750	3.172139	0.259461
C	-5.813330	3.309030	-0.066963
C	-7.991714	4.299671	0.558751

C	-9.310342	4.132424	0.876737
C	-9.888524	2.822733	0.917699
C	-9.131212	1.719575	0.639233
C	-1.459655	1.336829	-1.356609
C	-0.654174	0.235139	-1.617110
C	-1.227921	-1.091821	-1.533153
C	-2.573177	-1.228168	-1.211858
C	-0.377618	-2.221860	-1.766298
C	0.947254	-2.051902	-2.056648
C	1.515513	-0.740369	-2.132668
C	0.739063	0.366124	-1.927720
C	-5.311979	-1.571263	-0.490975
H	-7.384745	-0.252527	0.044344
H	-5.368336	4.299719	-0.098882
H	-7.549238	5.292440	0.526226
H	-9.932154	4.994691	1.101607
H	-10.939090	2.713929	1.173083
H	-9.567187	0.723854	0.669441
H	-1.026148	2.327912	-1.414746
H	-3.004545	-2.222286	-1.151966
H	-0.809635	-3.217043	-1.695303
H	1.585298	-2.915033	-2.220577
H	2.576034	-0.632023	-2.340176
H	1.171183	1.362375	-1.967993
C	-5.723317	-2.718859	-0.391672
Si	-6.175975	-4.488256	-0.207326
C	-4.854491	-5.322600	0.906178
C	-3.455699	-4.687841	0.787930
C	-4.781910	-6.846188	0.680050
C	-7.901755	-4.546467	0.602141
C	-7.936271	-3.813114	1.955526

C	-8.456140	-5.976994	0.735876
C	-6.219273	-5.261747	-1.951347
C	-7.375855	-4.693044	-2.795187
C	-4.877734	-5.090568	-2.689703
H	-5.202697	-5.154216	1.936806
H	-3.478851	-3.613310	0.992950
H	-3.035960	-4.816492	-0.217649
H	-2.754582	-5.156470	1.492811
H	-5.755327	-7.335673	0.798475
H	-4.089492	-7.313216	1.393453
H	-4.416720	-7.081648	-0.326941
H	-8.554223	-3.994147	-0.090801
H	-7.311077	-4.320587	2.701452
H	-8.957644	-3.782817	2.358913
H	-7.577592	-2.780916	1.869999
H	-9.486205	-5.963859	1.116996
H	-7.860863	-6.569992	1.441601
H	-8.465360	-6.510771	-0.222161
H	-6.396407	-6.339230	-1.809795
H	-7.381562	-5.138051	-3.799474
H	-7.276505	-3.607270	-2.918859
H	-8.355313	-4.889252	-2.343304
H	-4.925460	-5.531146	-3.694861
H	-4.048245	-5.572362	-2.159780
H	-4.625715	-4.029227	-2.806676

NC3 Cartesian coordinates:

C	-6.25641	8.78802	-3.07978
C	-6.90340	7.78666	-2.80540
C	-7.61640	6.63697	-2.39109

C	-6.91719	5.55813	-1.76499
C	-7.66527	4.44136	-1.21736
C	-9.08655	4.40304	-1.34546
C	-9.77887	5.45087	-2.02190
C	-9.03604	6.58468	-2.54324
C	-6.96466	3.40834	-0.56068
C	-5.57396	3.40365	-0.46291
C	-4.81864	4.49255	-1.05289
C	-5.50792	5.54166	-1.66479
C	-3.38924	4.44641	-0.98947
C	-2.74453	3.40385	-0.37072
C	-3.49091	2.34345	0.23236
C	-4.86456	2.34433	0.18932
C	-9.74705	7.61756	-3.19144
C	-11.13359	7.57660	-3.35279
C	-11.87190	6.44008	-2.83599
C	-11.18005	5.41835	-2.18675
C	-13.29245	6.40861	-3.00598
C	-13.94692	7.43208	-3.64450
C	-13.22098	8.55404	-4.15340
C	-11.85596	8.62240	-4.01302
C	-9.79834	3.31869	-0.79086
H	-7.53366	2.57869	-0.13096
H	-4.94412	6.37241	-2.09998
H	-2.82067	5.26658	-1.43976
H	-1.65136	3.38410	-0.32517
H	-2.95759	1.52459	0.72454
H	-5.44265	1.53007	0.63918
H	-9.18703	8.47329	-3.58156
H	-11.73525	4.56238	-1.79057
H	-13.84435	5.54843	-2.61167

H	-15.03388	7.39462	-3.76772
H	-13.76325	9.36074	-4.65593
H	-11.29326	9.47798	-4.40196
C	-10.41082	2.38774	-0.26824
Si	-11.27372	0.97039	0.51066
C	-10.53274	-0.65109	-0.16737
C	-10.04359	-0.53846	-1.61989
C	-11.51675	-1.82442	-0.01847
C	-10.97793	1.10921	2.38526
C	-9.48003	1.11510	2.72200
C	-11.71481	0.01552	3.17195
C	-13.12131	1.15300	0.10860
C	-13.68364	2.43977	0.73127
C	-13.38017	1.13702	-1.40616
H	-9.65004	-0.86305	0.46903
H	-9.31401	0.27985	-1.74367
H	-10.88430	-0.33810	-2.30745
H	-9.56888	-1.48619	-1.94074
H	-11.83724	-1.97447	1.02707
H	-11.04968	-2.76550	-0.36835
H	-12.42164	-1.65718	-0.62843
H	-11.39804	2.08975	2.68416
H	-9.32335	1.22895	3.81135
H	-8.95376	1.93927	2.21020
H	-9.00292	0.16424	2.41924
H	-12.79695	0.00005	2.94789
H	-11.59822	0.17652	4.26045
H	-11.30462	-0.98405	2.94266
H	-13.63691	0.28468	0.56536
H	-14.76415	2.53937	0.51318
H	-13.17373	3.32637	0.31295

H	-13.55406	2.46393	1.82733
H	-13.06008	0.18987	-1.87329
H	-12.82956	1.95776	-1.90006
H	-14.45749	1.27571	-1.61958
C	-3.76881	11.29228	-4.59743
C	-4.60172	10.00096	-4.49443
C	-5.49110	10.01771	-3.22670
C	-6.43157	11.24779	-3.29232
C	-5.59846	12.53554	-3.38123
C	-4.72598	12.49723	-4.64348
C	-2.87842	11.41507	-3.34715
C	-3.73714	11.45708	-2.05923
C	-4.70440	12.66587	-2.13859
C	-4.57695	10.15696	-1.98256
H	-3.12066	11.26163	-5.53474
H	-3.93023	9.12314	-4.45967
H	-5.24199	9.89559	-5.38916
H	-7.07372	11.26120	-2.39334
H	-7.08910	11.15318	-4.17564
H	-6.28093	13.40396	-3.42534
H	-4.13680	13.42962	-4.72832
H	-5.36410	12.42459	-5.54489
H	-2.26868	12.33447	-3.41049
H	-2.18368	10.55681	-3.29530
C	-2.90677	11.51580	-0.86273
H	-4.11503	13.59905	-2.18392
H	-5.31784	12.69898	-1.22074
H	-3.90226	9.28521	-1.92388
H	-5.19583	10.16867	-1.06949
C	-2.24564	11.46264	0.16517
C	-1.58512	11.29282	1.40554

C	-0.78042	12.33190	1.95861
C	-0.12992	12.13372	3.24361
C	-0.29365	10.89932	3.93991
C	-1.11192	9.86416	3.39507
C	-1.75913	10.06069	2.10930
C	0.65461	13.17856	3.77685
C	0.82427	14.39212	3.10926
C	0.18250	14.58826	1.82217
C	-0.59796	13.55978	1.28799
C	0.36920	15.83547	1.14629
C	1.13538	16.83203	1.70292
C	1.76535	16.63885	2.97296
C	1.61466	15.45330	3.65181
C	-2.55211	9.01623	1.58607
C	-2.73715	7.81029	2.26468
C	-2.10333	7.62248	3.55635
C	-1.31192	8.64734	4.08020
C	-2.30715	6.38789	4.24862
C	-3.07486	5.39001	3.69465
C	-3.69073	5.56978	2.41514
C	-3.52932	6.74829	1.72662
C	0.36656	10.68056	5.16939
H	1.14123	13.02644	4.74553
H	-1.08941	13.70672	0.32099
H	-0.11736	15.98099	0.17580
H	1.27062	17.78159	1.17596
H	2.37022	17.44438	3.40122
H	2.09630	15.29798	4.62331
H	-3.04096	9.16278	0.61907
H	-0.82940	8.50637	5.05178
H	-1.83284	6.25470	5.22742

H	-3.22130	4.44794	4.23192
H	-4.28287	4.75924	1.97927
H	-4.00633	6.88904	0.75093
C	0.91186	10.45705	6.25046
Si	1.67689	10.04841	7.86684
C	1.13737	11.35444	9.14742
C	0.92084	12.74103	8.52062
C	2.12739	11.44328	10.32169
C	1.07346	8.30585	8.33659
C	-0.45599	8.21569	8.42248
C	1.72706	7.80598	9.63426
C	3.56496	10.01767	7.64149
C	3.98087	8.85771	6.72380
C	4.10772	11.35042	7.10697
H	0.16233	11.00812	9.54556
H	0.16485	12.71040	7.71859
H	1.85728	13.12557	8.07827
H	0.59029	13.46597	9.28892
H	3.11589	11.79213	9.97430
H	2.27249	10.47250	10.82564
H	1.76708	12.16815	11.07641
H	1.40734	7.64983	7.50833
H	-0.93563	8.52838	7.47833
H	-0.84534	8.86158	9.23061
H	-0.77367	7.17922	8.64692
H	1.43268	6.75913	9.84168
H	1.40441	8.41495	10.49796
H	2.82996	7.84472	9.58564
H	4.00008	9.84163	8.64647
H	5.07990	8.83720	6.59237
H	3.52669	8.97379	5.72268

H	3.66823	7.87954	7.12831
H	5.20267	11.29101	6.95314
H	3.90815	12.18574	7.80014
H	3.64428	11.59562	6.13430

Supplementary References

1. Klaić, L., Alešković, M., Veljković, J., Mlinarić-Majerski, K. Carbenes in polycyclic systems: Generation and fate of potential adamantane-1,3-dicarbenes. *J. Phys. Org. Chem.* **21**, 299-305 (2008).
2. Lehnherr, D., Murray, A.H., McDonald, R., Tykwinski, R.R. A modular synthetic approach to conjugated pentacene di-, tri-, and tetramers. *Angew. Chem. Int. Ed.* **49**, 6190-6194 (2010).
3. Broxterman, Q.B., Hogeveen, H., Kingma, R.F. Preparation of 1,3-dialkynyladamantanes and their aluminum bromide-mediated cycloaddition adducts. *Tetrahedron Lett.* **27**, 1055-1058 (1986).
4. Archibald, T.G., Malik, A.A., Baum, K., Unroe, M.R. Thermally stable acetylenic adamantane polymers. *Macromolecules* **24**, 5261-5265 (1991).
5. Vincett, P.S., Voigt, E.M., Rieckhoff, K.E. Phosphorescence and fluorescence of phthalocyanines. *J. Chem. Phys.* **55**, 4131-4140 (1971).
6. Zhao, Y., Truhlar, D.G. Density functional for spectroscopy: No long-range self-interaction error, good performance for rydberg and charge-transfer states, and better performance on average than B3LYP for ground states. *J. Phys. Chem. A* **110**, 13126-13130 (2006).

7. Zhao, Y., Truhlar, D.G. The M06 suite of density functionals for main group thermochemistry, thermochemical kinetics, noncovalent interactions, excited states, and transition elements: Two new functionals and systematic testing of four M06-class functionals and 12 other functionals. *Theor. Chem. Acc.* **120**, 215-241 (2008).

8. Smith, M.B., Michl, J. Singlet fission. *Chem. Rev.* **110**, 6891-6936 (2010).

9. Nakamura, H., Truhlar, D.G. The direct calculation of diabatic states based on configurational uniformity. *J. Chem. Phys.* **115**, 10353-10372 (2001).

10. Nakamura, H., Truhlar, D.G. Direct diabatization of electronic states by the fourfold way. II. Dynamical correlation and rearrangement processes. *J. Chem. Phys.* **117**, 5576-5593 (2002).

11. Ruedenberg, K., Atchity, G.J. A quantum chemical determination of diabatic states. *J. Chem. Phys.* **99**, 3799-3803 (1993).

12. Atchity, G.J., Ruedenberg, K. Determination of diabatic states through enforcement of configurational uniformity. *Theor. Chem. Acc.* **97**, 47-58 (1997).

13. Zeng, T., Hoffmann, R., Ananth, N. The low-lying electronic states of pentacene and their roles in singlet fission. *J. Am. Chem. Soc.* **136**, 5755-5764 (2014).

14. Zirzlmeier, J., *et al.* Solution-based intramolecular singlet fission in cross-conjugated pentacene dimers. *Nanoscale* **8**, 10113-10123 (2016).

15. Smith, M.B., Michl, J. Recent advances in singlet fission. *Annu. Rev. Phys. Chem.* **64**, 361-386 (2013).

16. Berkelbach, T.C., Hybertsen, M.S., Reichman, D.R. Microscopic theory of singlet exciton fission. II. Application to pentacene dimers and the role of superexchange. *J. Chem. Phys.* **138**, 114103 (2013).

17. Nitzan, A. *Chemical dynamics in condensed phases: Relaxation, transfer, and reactions in condensed molecular systems*. Oxford University Press (2014).

18. Swenberg, C.E., Geacintov, N.E. In: *Organic molecular photophysics, vol 1* (eds Birks JB). Wiley (1973).

19. Benk, H., Sixl, H. Theory of 2 coupled triplet-states: Application to bicarbene structures. *Mol. Phys.* **42**, 779-801 (1981).

20. Burdett, J.J., Piland, G.B., Bardeen, C.J. Magnetic field effects and the role of spin states in singlet fission. *Chem. Phys. Lett.* **585**, 1-10 (2013).
